# Influence of periodic pulse intake on the ventilation efficiency of positive pressure explosion-proof robot

**DOI:** 10.1038/s41598-024-52011-9

**Published:** 2024-01-16

**Authors:** Ming Fang, Xufeng Chu, Liang Yu, Yu Fang, Liangliang Hou, Xu Cheng, Junlong Wang

**Affiliations:** 1 School of Artificial Intelligence, Anhui Polytechnic University, Wuhu, 241000 China; 2 R&D Center, Efort Intelligent Equipment Co., Ltd, Wuhu, 241007 China; 3 School of Mechanical Engineering, Anhui Polytechnic University, Wuhu, 241000 China

**Keywords:** Mechanical engineering, Fluid dynamics

## Abstract

The ventilation work is an important step to be completed before the start of the positive pressure explosion-proof robot. The existing explosion-proof technology uses constant pressure inflation, which will cause explosive gas to accumulate in the corner area of the cavity for a long time. In order to solve this problem, a ventilation method with periodic pulse intake is proposed. Based on the finite element method, the cleaning and ventilation process of the positive pressure explosion-proof robot is simulated and analyzed. The concentration of explosive gas in the robot cavity with time under constant pressure intake and pulse intake with different periods and amplitudes is compared. The simulation results show that the pulse intake is beneficial to the ventilation of the corner position. The period and amplitude of the pulse intake has an effect on the ventilation efficiency, when the period is the same, the greater the amplitude of the pulse intake, the higher the ventilation efficiency; when the amplitude is the same, the smaller the period of the pulse intake, the higher the ventilation efficiency. After experimental verification, the validity of the simulation results is proved. This study helps to improve the ventilation efficiency of positive-pressure explosion-proof robots and provides guidance for practical applications.

## Introduction

With the continuous development of modern science and technology, the level of industrial automation is increasing day by day. Industrial robots are widely used in various fields, among which spraying robots are mainly used in product surface coating operations. During the working process of the spraying robot, the sprayed paint volatilizes combustible gases such as benzene and xylene. If the electrical components inside the spraying robot produce sparks or the heat of the cavity does not dissipate in time to raise the temperature, it will ignite the volatile flammable gas and cause an explosion. Therefore, in order to ensure the safety of the spraying process, the spraying robot itself must have explosion-proof performance^1^.

Currently, the most used explosion-proof technology in the field of spraying robots is the positive pressure explosion-proof design. Before the robot starts, the protective gas (pure air or inert gas) is filled into the positive pressure cavity, and the explosive gas in the positive pressure cavity is discharged, so that the concentration of the explosive gas is reduced to below the safety value. This process is the process of cleaning and ventilation. During the operation of the robot, a small amount of protective gas needs to be continuously introduced into the positive pressure cavity to keep the pressure inside the cavity higher than the external environment and prevent explosive gases from entering the robot cavity, thus achieving the effect of explosion-proof, this process is the positive pressure maintenance process. Compared to other types of explosion-proof technology, positive pressure explosion-proof requires less mechanical strength, and the weight of positive pressure shell is smaller under the same volume^2^. In order to reduce the weight of the explosion-proof shell, Rong et al.^3^ used a positive pressure explosion-proof design method. There is a set of pressure and flow control system in the positive pressure cavity. The system collects the pressure difference in the cavity through the differential pressure sensor, and feeds back to the controller. The controller controls the on–off of the solenoid valve, so that the air pressure in the positive pressure cavity is kept within a safe range. Wang et al.^4^ similarly proposed the use of positive pressure explosion-proof technology to reduce the weight of the robot housing. Liu et al.^5^ applied the multi-component fluid theory to the positive pressure explosion-proof motor for the first time. The finite element method was used to simulate and analyze the multi-component transient concentration field during the purge process. Through the concentration cloud diagram, it can be known whether there is a purge dead angle inside the motor, which enhances the safety performance. Zhu et al.^6^ used the design of the static positive pressure main box in the coal mine rescue robot, and calculated the explosion-proof effectiveness of the box to reduce the wall thickness of the box and reduce the quality of the whole robot. In order to reduce the weight and improve the strength of the robot shell, the HADES detection and rescue robot developed by Victoria University in Wellington, New Zealand, uses a 2 mm thick glass fiber material, the robot not only meets the explosion-proof requirements in zone 1 environment, but also meets the waterproof and dust-proof level of IP67^7^. The positive pressure ventilation explosion-proof system designed by Nie et al.^8^ for spraying robot is composed of robot cavity, positive pressure and flow picking system, pressure reducing valve, catheter, explosion-proof solenoid valve, throttle valve and PLC controller. When the robot system is running, the PLC controller can identify the positive pressure and flow signal in the robot cavity, so as to control the on–off of the solenoid valve, realize the switching between the two working states of the explosion-proof system, and ensure the normal operation of the robot.

The above people have done a lot of research on the positive pressure explosion-proof technology, mainly the lightweight design of the positive pressure shell, or the optimization of the pressure and flow control system. However, the existing technology in the ventilation of the use of constant pressure to the cavity filled with gas, the cavity will form a stable flow field, the corners of the location of the stagnant area of the air flow, resulting in the accumulation of explosive gases, which is not conducive to the ventilation effect of the corners of the cavity location. Based on the existing positive pressure explosion-proof structure of spraying robot, this paper adopts the ventilation method of periodic pulse intake, and analyzes the influence of the period and amplitude of pulse intake on the ventilation efficiency. It is hoped to provide guidance for practical application and ensure that the cleaning and ventilation work can be completed in the least time before the robot is opened to achieve effective explosion-proof.

## Simulation model and simulation parameters

### Mathematical model

When the fluid moves, it is subject to the laws of physical conservation. The basic conservation laws include the laws of mass conservation, momentum conservation and energy conservation. If the flow field contains different components, the system also has to obey the law of conservation of components. If the flow is in a turbulent state, the system must obey the turbulence equation^9–14^.

*Mass conservation equation*

1 $$\begin{array}{c}\frac{\partial \rho }{\partial t}+u\frac{\partial \left(\rho u\right)}{\partial x}+v\frac{\partial \left(\rho v\right)}{\partial y}+w\frac{\partial \left(\rho w\right)}{\partial z}=0\end{array}$$

where ρ is the density, t is the time, u, v and w are the velocity components in the x, y and z directions.

*Momentum conservation equation*

2 $$\begin{array}{c} \left\{\begin{array}{c}\frac{\partial \left(\mathrm{\rho u}\right)}{\partial {\text{t}}}+\frac{\partial \left(\mathrm{\rho uu}\right)}{\partial {\text{x}}}+\frac{\partial \left(\mathrm{\rho uv}\right)}{\partial {\text{y}}}+\frac{\partial \left(\mathrm{\rho uw}\right)}{\partial {\text{z}}}=-\frac{\partial {\text{p}}}{\partial {\text{x}}}+\frac{\partial {\uptau }_{{\text{xx}}}}{\partial {\text{x}}}+\frac{\partial {\uptau }_{{\text{yx}}}}{\partial {\text{y}}}+\frac{\partial {\uptau }_{{\text{zx}}}}{\partial {\text{z}}}+{{\text{F}}}_{{\text{x}}}\\ \frac{\partial \left(\mathrm{\rho v}\right)}{\partial {\text{t}}}+\frac{\partial \left(\mathrm{\rho vu}\right)}{\partial {\text{x}}}+\frac{\partial \left(\mathrm{\rho vv}\right)}{\partial {\text{y}}}+\frac{\partial \left(\mathrm{\rho uw}\right)}{\partial {\text{z}}}=-\frac{\partial {\text{p}}}{\partial {\text{y}}}+\frac{\partial {\uptau }_{{\text{xy}}}}{\partial {\text{x}}}+\frac{\partial {\uptau }_{{\text{yy}}}}{\partial {\text{y}}}+\frac{\partial {\uptau }_{{\text{zy}}}}{\partial {\text{z}}}+{{\text{F}}}_{{\text{y}}}\\ \frac{\partial \left(\mathrm{\rho w}\right)}{\partial {\text{t}}}+\frac{\partial \left(\mathrm{\rho wu}\right)}{\partial {\text{x}}}+\frac{\partial \left(\mathrm{\rho wv}\right)}{\partial {\text{y}}}+\frac{\partial \left(\mathrm{\rho ww}\right)}{\partial {\text{z}}}=-\frac{\partial {\text{p}}}{\partial {\text{x}}}+\frac{\partial {\uptau }_{{\text{xz}}}}{\partial {\text{x}}}+\frac{\partial {\uptau }_{{\text{yz}}}}{\partial {\text{y}}}+\frac{\partial {\uptau }_{{\text{zz}}}}{\partial {\text{z}}}+{{\text{F}}}_{{\text{z}}}\end{array}\right.\end{array}$$

where p is the pressure on the fluid micro element; ${\uptau }_{{\text{xx}}}$, ${\uptau }_{{\text{xy}}}$ and ${\tau }_{xz}$ are the components of the viscous stress τ along the x, y, and z directions; ${{\text{F}}}_{{\text{x}}}$, ${{\text{F}}}_{{\text{y}}}$, and ${{\text{F}}}_{{\text{z}}}$ are the volume forces on the fluid micro element.

*Energy conservation equation*

3 $$\begin{array}{c}\frac{\partial \left(\rho T\right)}{\partial t}+\frac{\partial \left(\rho uT\right)}{\partial x}+\frac{\partial \left(\rho vT\right)}{\partial y}+\frac{\partial \left(\rho wT\right)}{\partial z}=\frac{\partial }{\partial x}\left(\frac{k}{{c}_{p}}\frac{\partial T}{\partial x}\right)+\frac{\partial }{\partial y}\left(\frac{k}{{c}_{p}}\frac{\partial T}{\partial y}\right)+\frac{\partial }{\partial z}\left(\frac{k}{{c}_{p}}\frac{\partial T}{\partial z}\right)+{S}_{T}\end{array}$$

where ${c}_{p}$ is specific heat capacity; T is temperature; k is heat transfer coefficient of the fluid; ${S}_{T}$ is viscous dissipation term.

*Component conservation equation*

4 $$\begin{array}{c}\left\{\begin{array}{c}{\alpha }_{f}={\sum }_{i=1}^{n}{Y}_{i}{\alpha }_{i}\\ {\beta }_{f}={\sum }_{i=1}^{n}{Y}_{i}{\beta }_{i}\\ {\sum }_{i=1}^{n}{Y}_{i}=1\end{array}\right.\end{array}$$

where n is the component fraction, ${Y}_{i}$ is the mass fraction of each component fluid; ${\alpha }_{i}$ is the density of each component fluid; ${\alpha }_{f}$ is the overall density; ${\beta }_{i}$ is the dynamic viscosity of each component fluid; ${\beta }_{f}$ is the overall dynamic viscosity; where n = 3(oxygen, nitrogen, helium); the densities of oxygen, nitrogen and helium are 1.43 kg/m^3^, 1.25 kg/m^3^ and 0.179 kg/m^3^, respectively. The dynamic viscosities of oxygen, nitrogen and helium are 2.06 × 10^−5^ kg/m s, 1.78 × 10^−5^ kg/m s and 1.89 × 10^−5^ kg/m s, respectively.

*Turbulence equation*

5 $$\begin{array}{c}\left\{\begin{array}{c}\frac{\partial \left(\rho k\right)}{\partial t}+\frac{\partial \left(\rho k{u}_{i}\right)}{\partial {x}_{i}}=\frac{\partial }{\partial {x}_{j}}\left[\left(\mu +\frac{{\mu }_{t}}{{\sigma }_{k}}\right)\frac{\partial k}{\partial {x}_{j}}\right]+{G}_{k}+{G}_{b}-\rho \varepsilon -{Y}_{M}+{S}_{k}\\ \frac{\partial \left(\rho \varepsilon \right)}{\partial t}+\frac{\partial \left(\rho \varepsilon {u}_{i}\right)}{\partial {x}_{i}}=\frac{\partial }{\partial {x}_{j}}\left[\left(\mu +\frac{{\mu }_{t}}{{\sigma }_{\varepsilon }}\right)\frac{\partial \varepsilon }{\partial {x}_{j}}\right]+{G}_{1\varepsilon }\frac{\varepsilon }{k}\left({G}_{k}+{G}_{3\varepsilon }{G}_{b}\right)-{G}_{2\varepsilon }\rho \frac{{\varepsilon }^{2}}{k}+{S}_{\varepsilon }\end{array}\right.\end{array}$$

where k is the turbulent pulse kinetic energy; ${u}_{i}$ is the velocity in $i$ direction; ${x}_{i}$ and ${x}_{j}$ are the direction of Cartesian coordinate system; μ is dynamic viscosity; ε is the turbulent pulse kinetic energy dissipation rate; ${\mu }_{t}$ is the turbulent viscosity, which can be expressed as a function of k and ε, that is, ${\mu }_{t}=\rho {G}_{\mu }{\varepsilon }^{2}/k$; ${G}_{k}$ is the turbulent kinetic energy generation term caused by the average velocity gradient; ${G}_{b}$ is the turbulent kinetic energy generation term caused by buoyancy; ${\sigma }_{k}$ and ${\sigma }_{\varepsilon }$ are the Prandtl numbers corresponding to the turbulent pulse kinetic energy and the turbulent pulse kinetic energy dissipation rate, respectively, taking 1.0 and 1.3; ${G}_{1\varepsilon }$, ${G}_{2\varepsilon }$ and ${G}_{3\varepsilon }$ are empirical coefficients, which are 1.44, 1.92 and 1.44, respectively.

### Geometric model and mesh division

**Figure 1:**
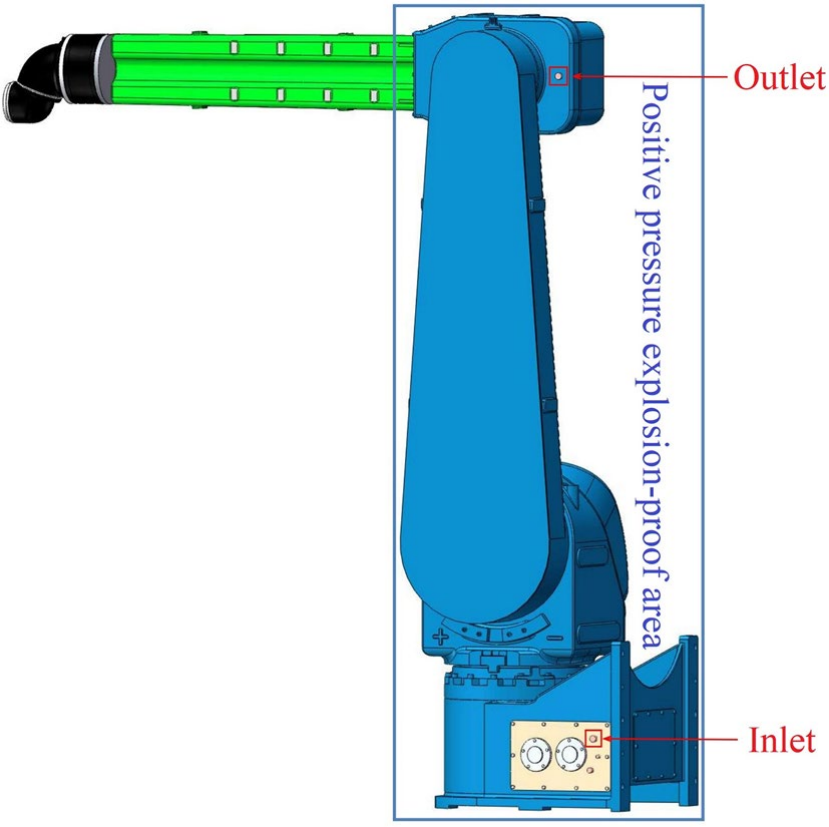
The structure diagram of the positive pressure explosion-proof robot.

In this paper, a positive pressure explosion-proof robot is selected as the research object, and its basic parameters are shown in Table 1. The volume of the positive pressure cavity of the robot is 0.1764 m^3^. The Inlet is on the side of the base with an inner diameter of 16 mm, and the Outlet is on the side of the top cavity with an inner diameter of 10 mm. The structure diagram of the positive pressure explosion-proof robot is shown in Fig. 1.

**Table 1:** The basic parameters of positive pressure explosion-proof robot.

Basic parameters	Numerical value
Rated voltage	380 V
Rated power	8KW
Rated frequency	50 Hz
Protection level	IP 65
Explosion proofing grade	Ex ib mb Px IIC T4 Gb
Working radius	2900 mm
Positive pressure cavity volume	0.1764 m^3^

**Figure 2:**
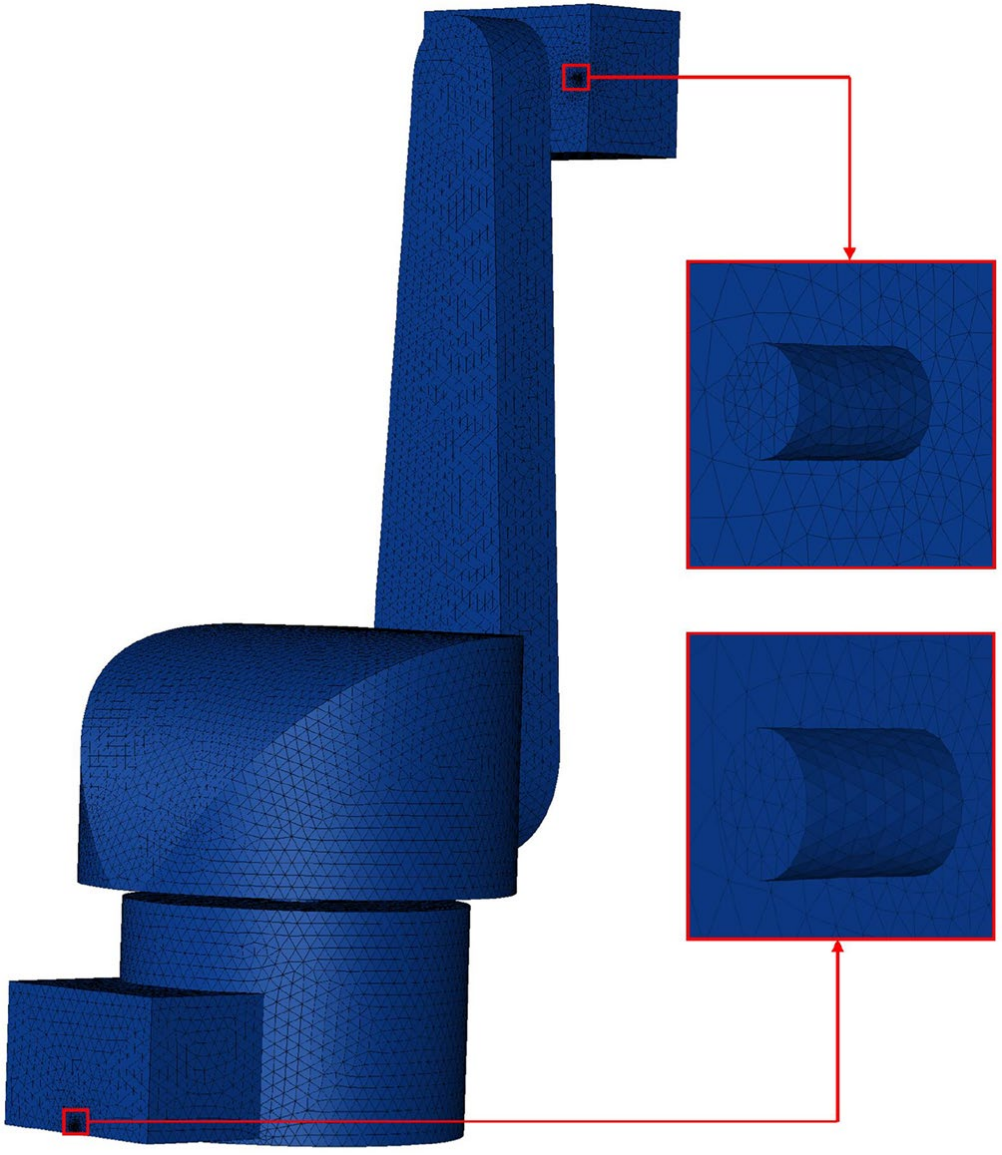
The gird mesh of model.

When the positive-pressure explosion-proof robot changes air, pure air is passed into the robot cavity from the inlet, and the explosive gases inside the cavity are discharged from the outlet. The flow field mainly exists inside the positive-pressure cavity of the robot, so this part is chosen as the flow field domain and divided into grids. Considering the influence of calculation speed and calculation accuracy, after comparison and trial calculation^11^, the final mesh unit is divided into 1,094,018. The gird mesh of model is shown in Fig. 2.

### Simulation parameters and strategies

*Simulation conditions*

1. The chemical reactions between multicomponent fluids are not considered.
2. The positive pressure cavity is under normal pressure, ignoring the influence of buoyancy and gravity in the fluid domain.
3. The flow of gas in the positive pressure cavity is a low-speed movement, so the gas is treated as an incompressible fluid.
4. ANSYS FLUENT is used for simulation calculation, and the pressure-based solver is selected as the solver.
5. The simulation calculation belongs to the transient flow field analysis. In order to improve the calculation accuracy and speed, PISO algorithm is selected.

*Boundary conditions*

**Figure 3:**
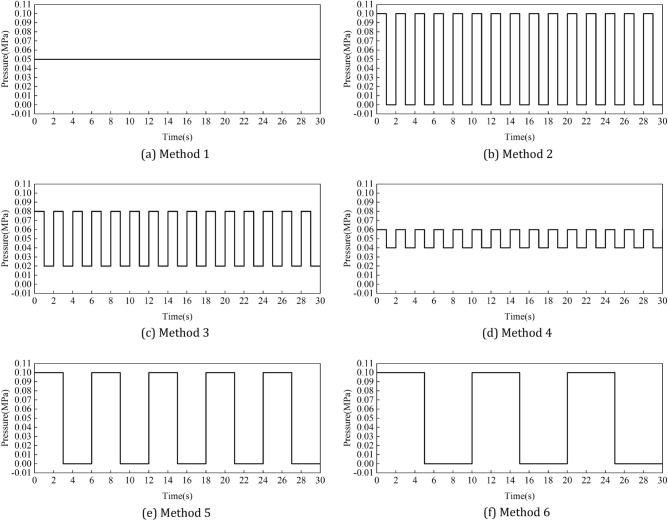
The periodic variation of intake pressure with time under different intake methods. (**a**) Method 1, (**b**) method 2, (**c**) method 3, (**d**) method 4, (**e**) method 5, (**f**) method 6.

1. The main explosive gases in the working environment of the spraying robot are toluene and xylene, according to international explosion-proof standards IEC 60079-2:2014, helium is used instead of explosive gas in ventilation simulation. (In the explosion-proof test, helium is often used to replace the explosive gas whose density is less than that of air. Because helium has low density and is inert gas, it has high safety)
2. The initial concentration percentage of helium in the positive pressure cavity of the robot is set to 75%.
3. Pure air (consisting of 21% oxygen and 79% nitrogen) is filled into the positive pressure chamber of the robot through the air inlet. The pressure inlet is adopted.
4. The pressure outlet is adopted.
5. The end condition of the simulation calculation is that the percentage of helium concentration in each region of the robot cavity cannot be higher than 1%.
6. A total of six intake methods were set up in the ventilation simulation. Method 1 was used as the reference method, and the intake pressure was constant at 0.05 MPa. In order to analyze the influence of pulse intake amplitude on ventilation efficiency, methods 2, 3 and 4 were set up in this study with pulse intake periods of 2 s and amplitudes of 0.05 MPa, 0.03 MPa and 0.01 MPa, respectively. In order to analyze the influence of pulse intake period on ventilation efficiency, method 2, method 5 and method 6 are set up in this study. The pulse intake amplitude is 0.05 MPa, and the period is 2 s, 6 s and 10 s respectively^15^. Figure 3 shows the periodic variation of intake pressure with time under different intake methods.

## Analysis of simulation results

**Figure 4:**
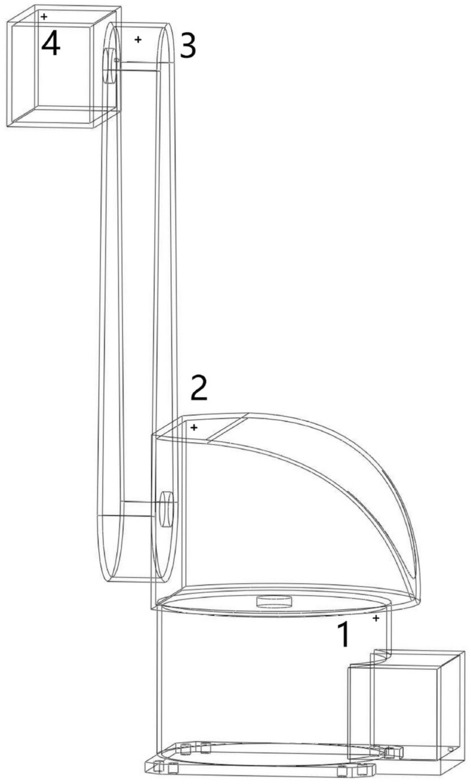
Location distribution of monitoring points.

In order to study the change of flow field and explosive gas distribution in the positive pressure cavity of the robot with time under different ventilation methods, ventilation simulation adopts transient process, the time step is 0.1 s, the maximum number of iterative steps per time step is 100, and the number of time steps is set to 1200. In the ventilation simulation, in order to facilitate the analysis of the simulation results in the explosion-proof area, a total of four monitoring points were established, all located in the corners of the robot cavity, i.e., the area where the adverse points of ventilation are most likely to occur. The specific location of each monitoring point is shown in Fig. 4.

### Comparative analysis of ventilation efficiency of constant pressure intake and pulse intake

**Figure 5:**
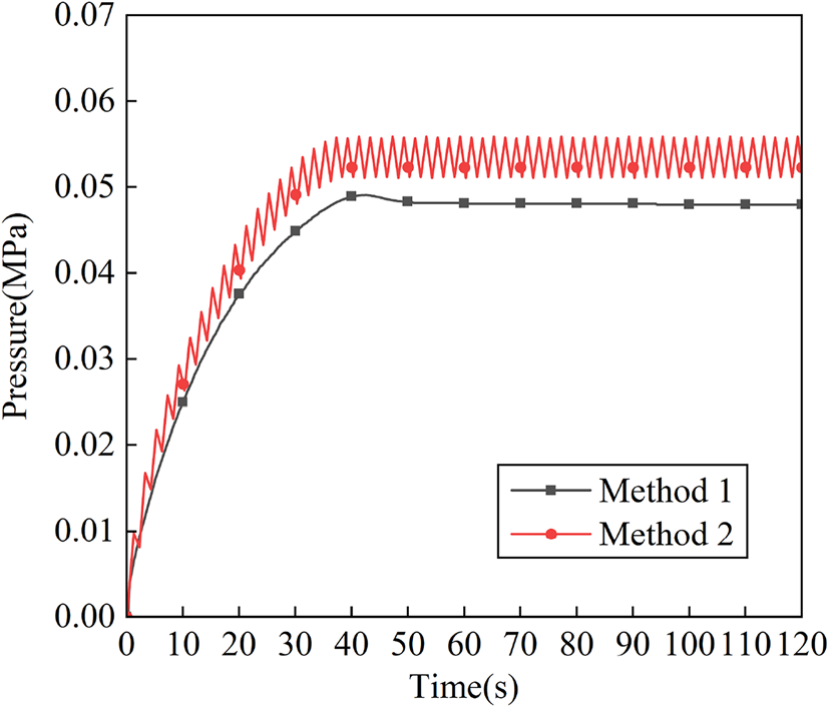
Variation of pressure in the positive pressure cavity with time of method 1 and method 2.

Taking Method 1 and Method 2 as examples, the influence of constant pressure intake and pulse intake of ventilation efficiency is compared and analyzed. Figure 5 shows the variation of the pressure inside the positive pressure cavity of the robot with time for the two intake methods. It can be seen that the pressure in the positive pressure cavity rises steadily during 0-40 s when the intake pressure is constant, and gradually tends to a stable value of 0.048 MPa after 40 s. When the pulse intake, the pressure in the positive pressure cavity fluctuates and rises during 0–40 s, and after 40 s the pressure oscillates continuously from 0.051 to 0.056 MPa, and the oscillation period is basically the same as that of the pulse intake. The space occupied by the airflow circuit of the pulse intake and the diffusion space of the helium gas create a repeated "push and pull" situation. The process of “push” is as follows: when the intake pressure changes from the minimum value to the maximum value, the air flow circuit gradually diffuses upward, and the diffusion space of helium is continuously reduced, and the helium along the way is transported to the outlet. The process of “pull” is as follows: when the intake pressure changes from the maximum value to the minimum value, the air flow circuit gradually shrinks, and the helium diffusion space gradually increases. A complete "push and pull" process is a ventilation process.

**Figure 6:**
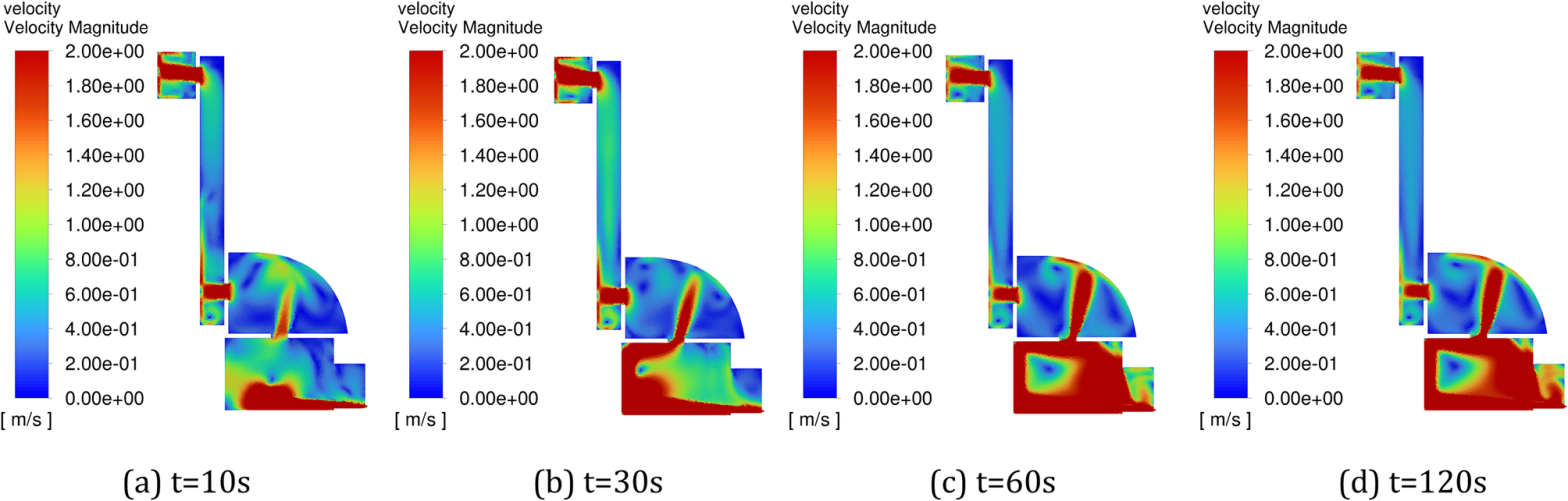
Variation of flow field with time in the positive pressure cavity of method 1. (**a**) t = 10 s, (**b**) t = 30 s, (**c**) t = 60 s, (**d**) t = 120 s.

**Figure 7:**
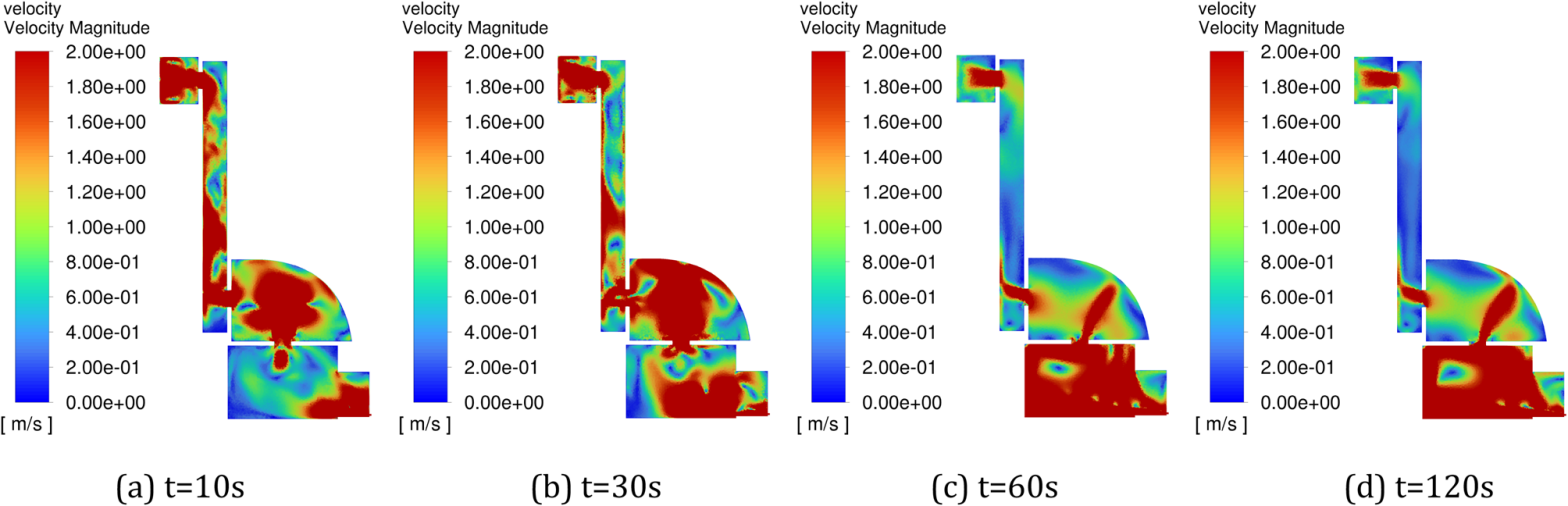
Variation of flow field with time in the positive pressure cavity of method 2. (**a**) t = 10 s, (**b**) t = 30 s, (**c**) t = 60 s, (**d**) t = 120 s.

Figure 6 shows the change of velocity field in the positive pressure cavity with time under the constant pressure intake method. It can be seen from the figure that when the inlet pressure is constant, the airflow continues to advance with time, and finally a stable flow field is formed in the cavity, and the gas velocity in the cavity (especially the corner position and the position far from the inlet) is slow. Figure 7 shows the change of velocity field in the positive pressure cavity with time under the pulse intake method. From the figure, it can be seen that the pulse inlet caused a large fluctuation of the flow field in the positive pressure chamber in the early stage, and the flow velocity of the gas increased, and the overall gas flow still flowed from the inlet to the outlet, and the direction was basically unchanged.

**Figure 8:**
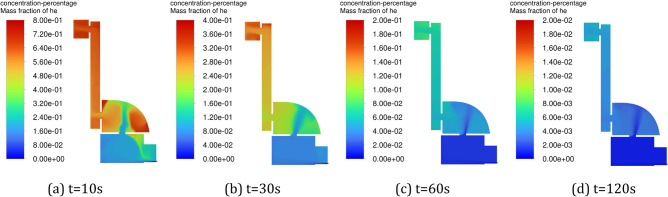
Variation of helium concentration with time in the positive pressure cavity of method 1. (**a**) t = 10 s, (**b**) t = 30 s, (**c**) t = 60 s, (**d**) t = 120 s.

**Figure 9:**
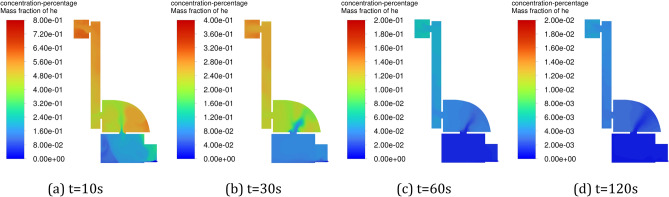
Variation of helium concentration with time in the positive pressure cavity of method 2. (**a**) t = 10 s, (**b**) t = 30 s, (**c**) t = 60 s, (**d**) t = 120 s.

Figure 8 shows the variation of helium concentration distribution in the positive pressure cavity with time when the inlet pressure is constant. From the figure, it can be seen that as the gas mainly flows in the main flow path, the flow velocity is slower in the corner position of the cavity, and with the gradual increase of the space occupied by the inlet gas flow, the diffusion space of helium is gradually compressed, and most of the helium accumulates in the corner stagnation area, resulting in a low ventilation efficiency. Figure 9 shows the variation of helium concentration distribution in the positive pressure cavity with time during pulse intake. As can be seen from the figure, due to the large fluctuation of the airflow, the doping effect of the pure air with the helium in the cavity is increasing, which accelerates the diffusion of helium and effectively avoids the buildup of the gas. The pulsed air intake makes the stagnation zone gradually shrink and the helium diffusion space expand upward, while driving the flow of airflow in the corner area, which effectively improves the ventilation efficiency in the corner area.

### Influence of amplitude variation of pulse intake on ventilation efficiency

**Figure 10:**
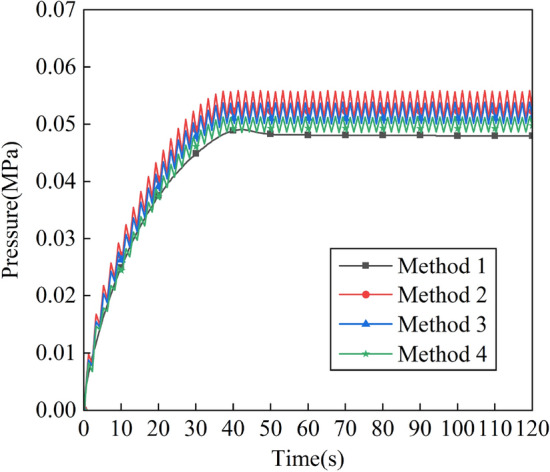
Variation of pressure in the positive pressure cavity with time for different intake amplitudes.

The variation of pressure in the positive pressure cavity with time for different intake amplitudes is shown in Fig. 10, and it can be seen that the intracavity pressure fluctuates and rises gradually in 0–40 s in methods 2, 3 and 4, with the fastest rise in method 2, the second fastest in method 3, and the slowest rise in method 4. After 40 s the pressure values in the cavity in Methods 2, 3 and 4 were constantly oscillating at 0.051–0.056 MPa, 0.05–0.054 MPa and 0.048–0.051 MPa, respectively.

**Figure 11:**
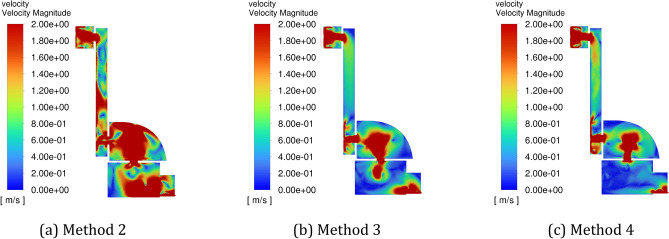
The schematic diagram of the flow field distribution in the cavity under different amplitude conditions at 30 s. (**a**) Method 2, (**b**) method 3, (**c**) method 4.

**Figure 12:**
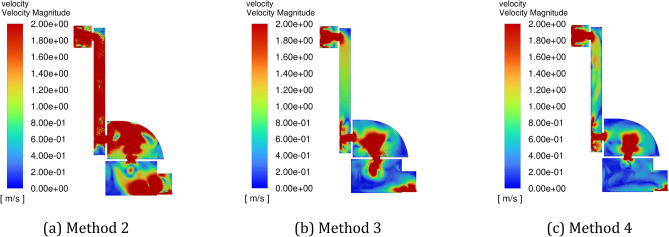
The schematic diagram of the flow field distribution in the cavity under different amplitude conditions at 31 s. (a) Method 2, (b) Method 3, (c) Method 4.

Figure 11 shows the flow field distribution in the cavity at 30 s (when inflating the positive pressure cavity with a valley value) with different amplitudes, and Fig. 12 shows the flow field distribution in the cavity at 30 s (when inflating the positive pressure cavity with a peak value) with different amplitudes. It can be seen from the figures that the gas flow rate in the chamber is fastest in method 2, second fastest in method 3 and slower in method 4. When the inlet pressure shifts from a valley to a peak, it increases the gas flow rate in the chamber, and since Method 2 has the largest amplitude, it has the most pronounced change in gas flow rate.

**Figure 13:**
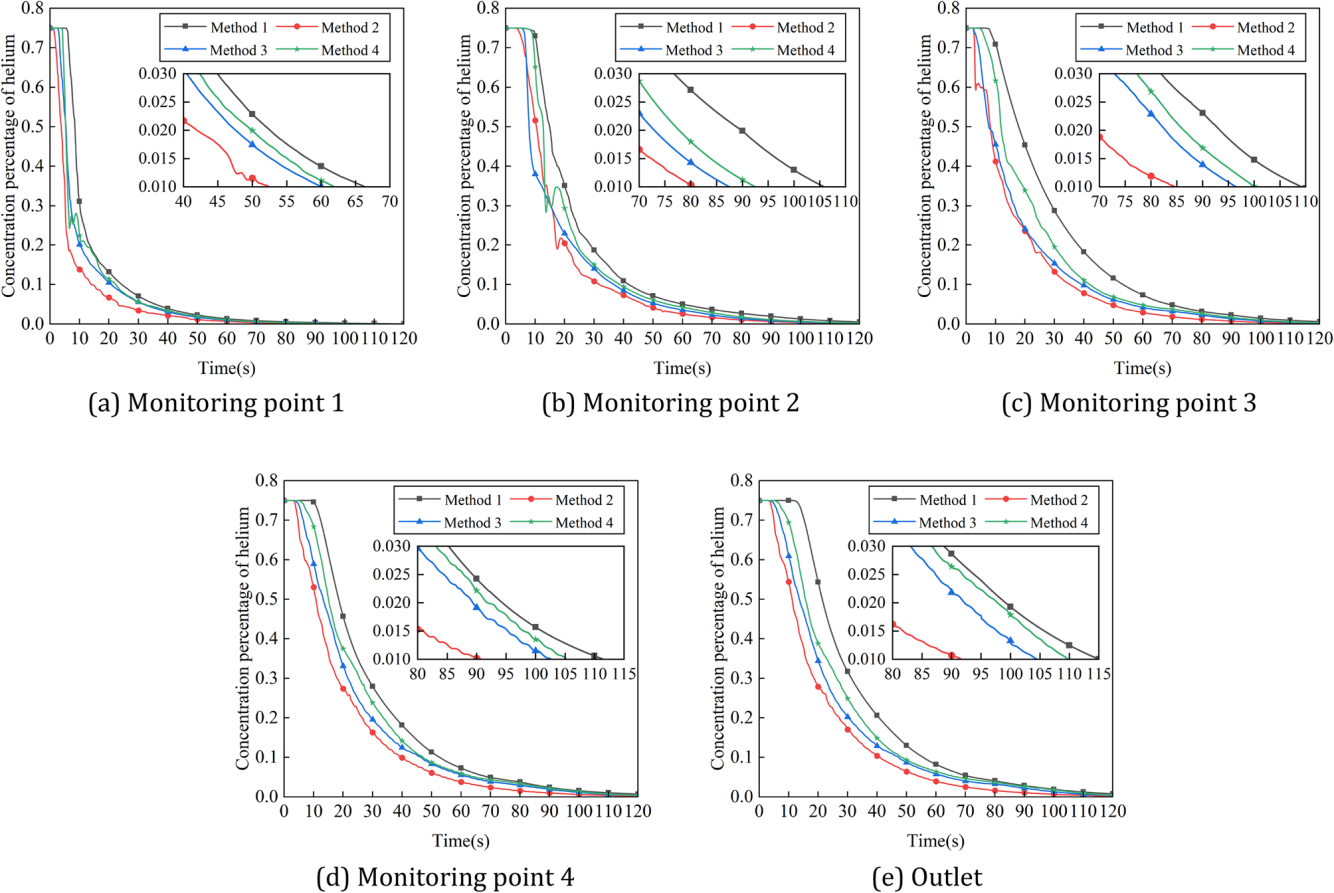
Variation of helium concentration in each monitoring area with time for different intake amplitudes. (**a**) Monitoring point 1, (**b**) monitoring point 2, (**c**) monitoring point 3, (**d**) monitoring point 4, (**e**) outlet.

The gas concentrations at the four corner locations and at the outlet of the intake method 1, method 2, method 3 and method 4 are compared over time, and the helium concentrations in the five monitoring areas over time are shown in Fig. 13. As can be seen from the graph, the helium concentration percentages in all the monitored areas begin to decrease in the order of Method 2, Method 3, Method 4, and Method 1, and Method 1's helium concentration percentage decreases less rapidly than the other three methods throughout the process. According to explosion protection standards, a helium concentration percentage of less than 1% in all areas of the positive pressure cavity is considered safe. At each monitoring area, the time taken for the percentage helium concentration to fall to a safe value was shortest in Method 2, followed by Methods 3 and 4, and the time taken for the percentage helium concentration to fall to a safe value was higher in Method 1 than in any of the other methods.

The process of decreasing the inlet pressure from the maximum to the minimum will provide a larger diffusion space for the helium, when the airflow forms an airflow circuit in order to deliver more helium to the return air outlet, when the inlet pressure changes from the minimum to the maximum, the airflow circuit gradually spreads upwards, continuously narrowing down the diffusion space for the helium, and at the same time, delivering helium along the way to the air outlet. When the pulse period is the same, the larger the amplitude of the pulse inlet, the stronger the disturbance of the airflow in the cavity, which can better drive the airflow in the whole cavity, and the ventilation efficiency of the positive-pressure explosion-proof robot will be higher. If the amplitude is reduced, the airflow circuit of the air intake fluctuates less, which is not conducive to the flow of gas in the corner areas of the robot cavity, and there will be no improvement in ventilation efficiency.

### Influence of periodic variation of pulse intake on ventilation efficiency

**Figure 14:**
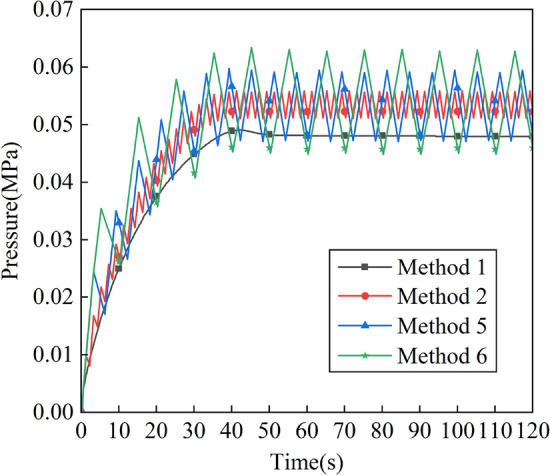
Variation of pressure in the positive pressure cavity with time for different intake periods.

The variation of intracavity pressure with time for different pulse cycle cases is shown in Fig. 14, and it can be seen that the intracavity pressure gradually fluctuates and rises within 0–40 in Method 2, Method 3 and Method 4. After 40 s the pressure values in the chamber in Methods 2, 3 and 4 were constantly oscillating at 0.051–0.056 MPa, 0.047–0.059 MPa and 0.045–0.061 MPa, respectively, and the period of change was basically the same as that of the pulsed air intake.

**Figure 15:**
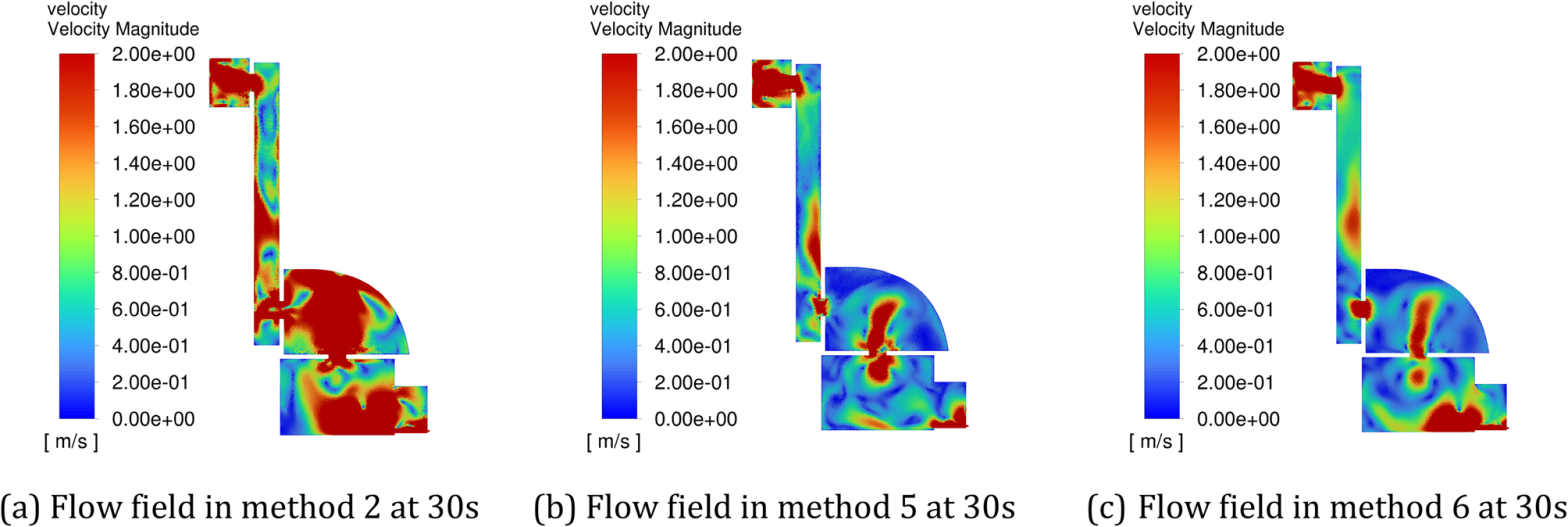
The schematic diagram of flow field distribution in the cavity under different pulse periods when the positive pressure cavity is inflated with a valley value. (**a**) Flow field in method 2 at 30 s. (**b**) Flow field in method 5 at 30 s. (**c**) Flow field in method 6 at 30 s.

**Figure 16:**
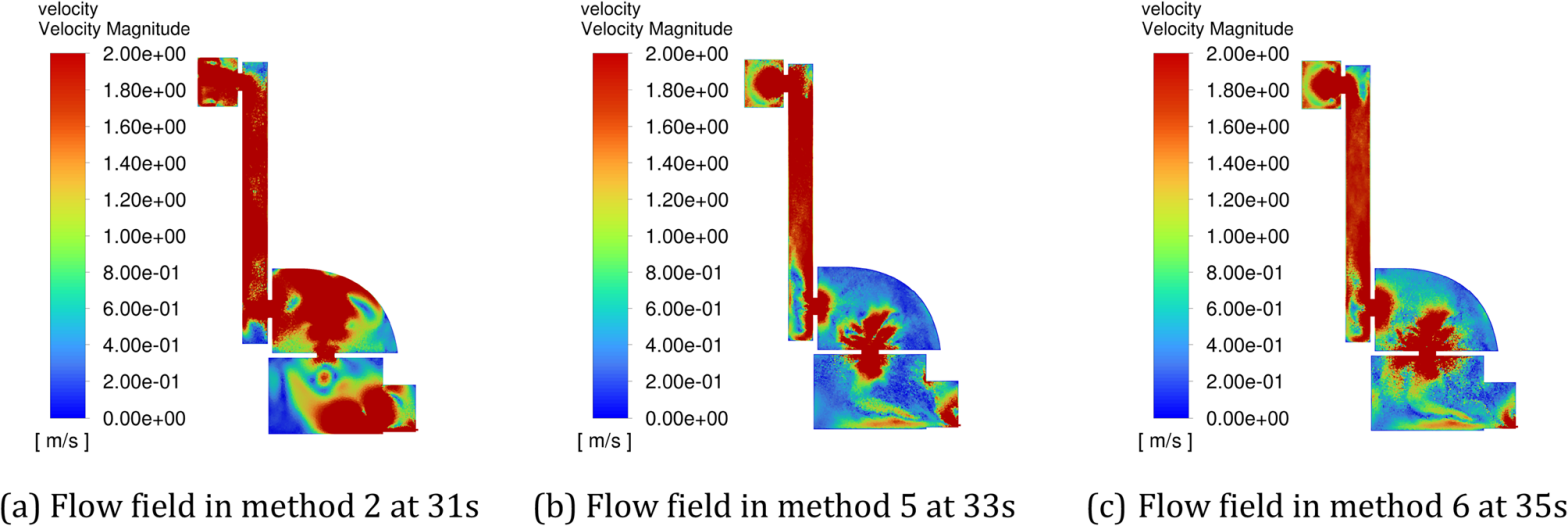
The schematic diagram of flow field distribution in the cavity under different pulse periods when the positive pressure cavity is inflated with a peak value. (**a**) Flow field in method 2 at 31 s. (**b**) Flow field in method 5 at 33 s. (**c**) Flow field in method 6 at 35 s.

Figure 15 shows the flow field distribution in the cavity at 30 s (when the positive pressure cavity is inflated with a valley value) with different periods, and Fig. 16 shows the flow field distribution in the cavity after 30 s with different amplitudes when the positive pressure cavity is inflated with a peak value. It can be seen from the figures that the gas flow rate in the chamber is the fastest in method 2. When the inlet pressure changes from a valley to a peak, it increases the gas flow rate in the chamber, and since Method 2 has the smallest period, it has the fastest frequency of gas flow rate change.

**Figure 17:**
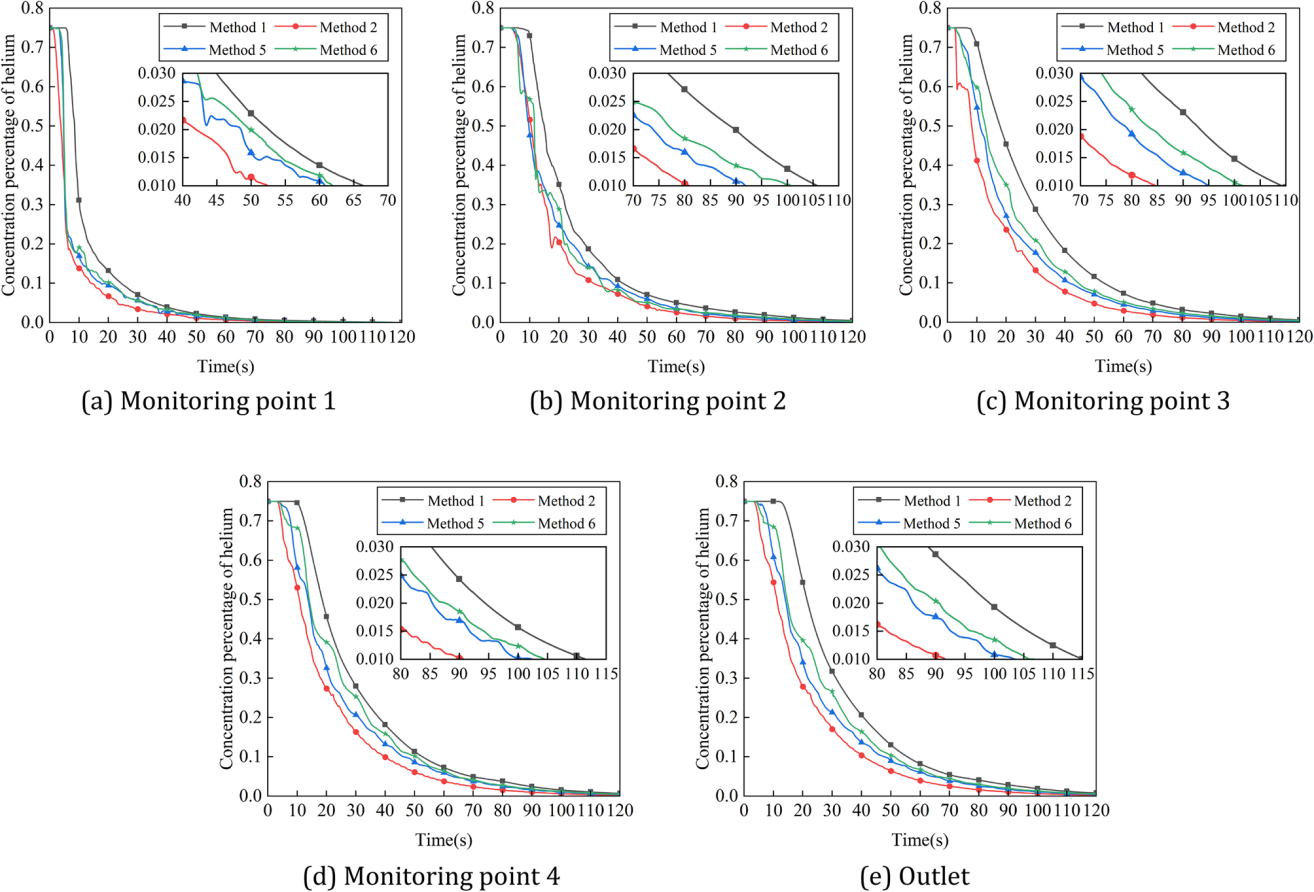
Variation of helium concentration in each monitoring area with time for different intake periods. (**a**) Monitoring point 1. (**b**) Monitoring point 2. (**c**) Monitoring point 3. (**d**) Monitoring point 4. (**e**) Outlet.

The gas concentrations at the four corner locations and at the outlet of the intake method 1, method 2, method 5 and method 6 are compared over time, and the helium concentrations in the five monitoring areas over time are shown in Fig. 17. It can be seen that at each monitoring area, the helium concentration decreases less rapidly with the constant pressure intake method than with each pulse intake method, and the ventilation efficiency of method 2 is higher than that of methods 5 and 6. In Method 1, the helium concentration curve in each monitoring area decreases smoothly, and in Methods 2, 5 and 6, the helium concentration at monitoring point 4 and at the outlet decreases in a regular fluctuation with essentially the same time and pulse period for each fluctuation.

In Method 2, Method 5 and Method 6, a perturbation is generated in the cavity every 2 s, every 6 s and every 10 s, respectively. When the amplitude is the same, the smaller the period of the pulse inlet, the more the number of disturbances of the gas flow in the cavity, which can increase the uneven distribution of the gas flow rate in the cavity, drive the gas flow in the gas stagnation area, and thus drive the helium to diffuse to the air outlet, so the ventilation efficiency will be higher.

## Experiment

### Experimental platform construction

**Figure 18:**
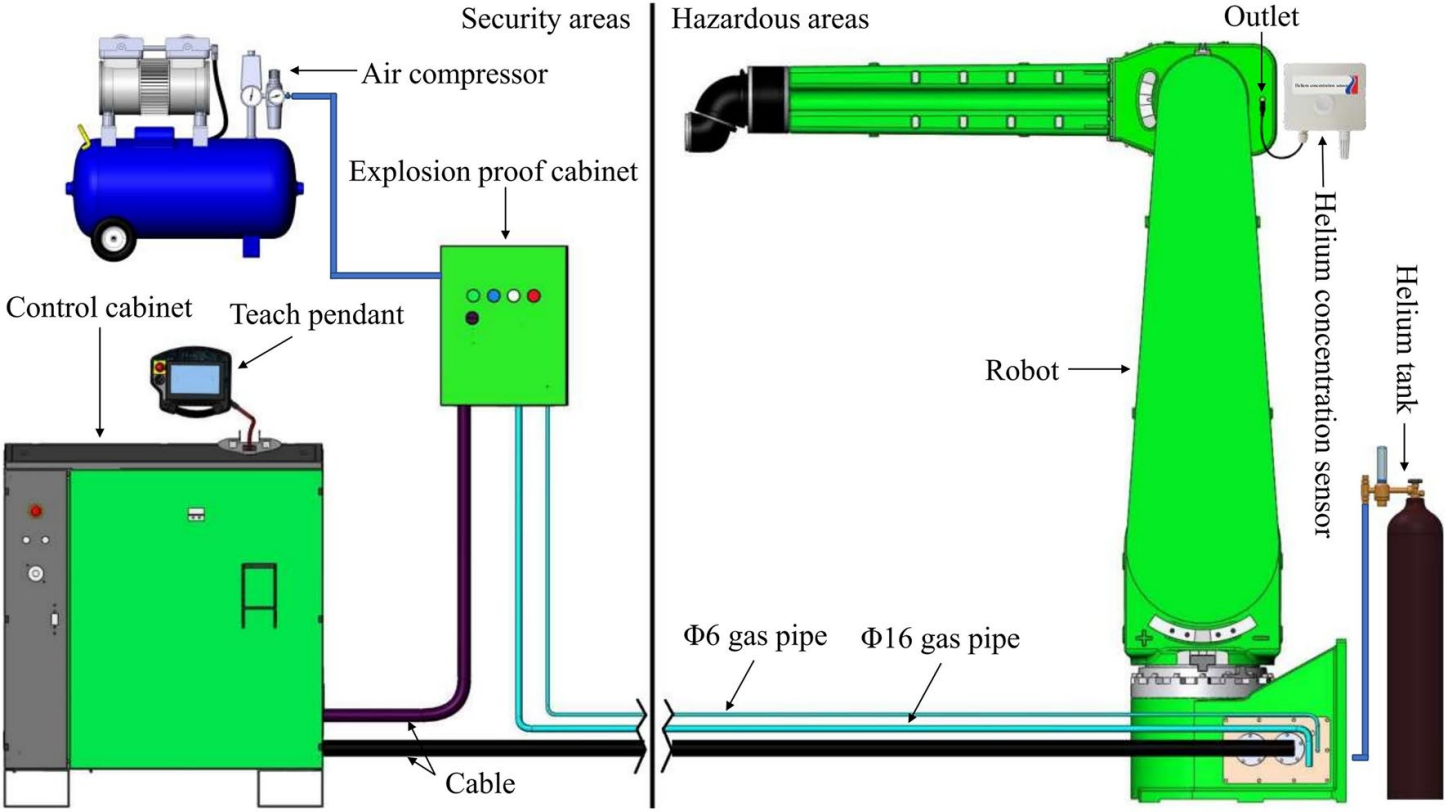
Schematic diagram of the positive pressure explosion-proof robot experimental platform.

**Figure 19:**
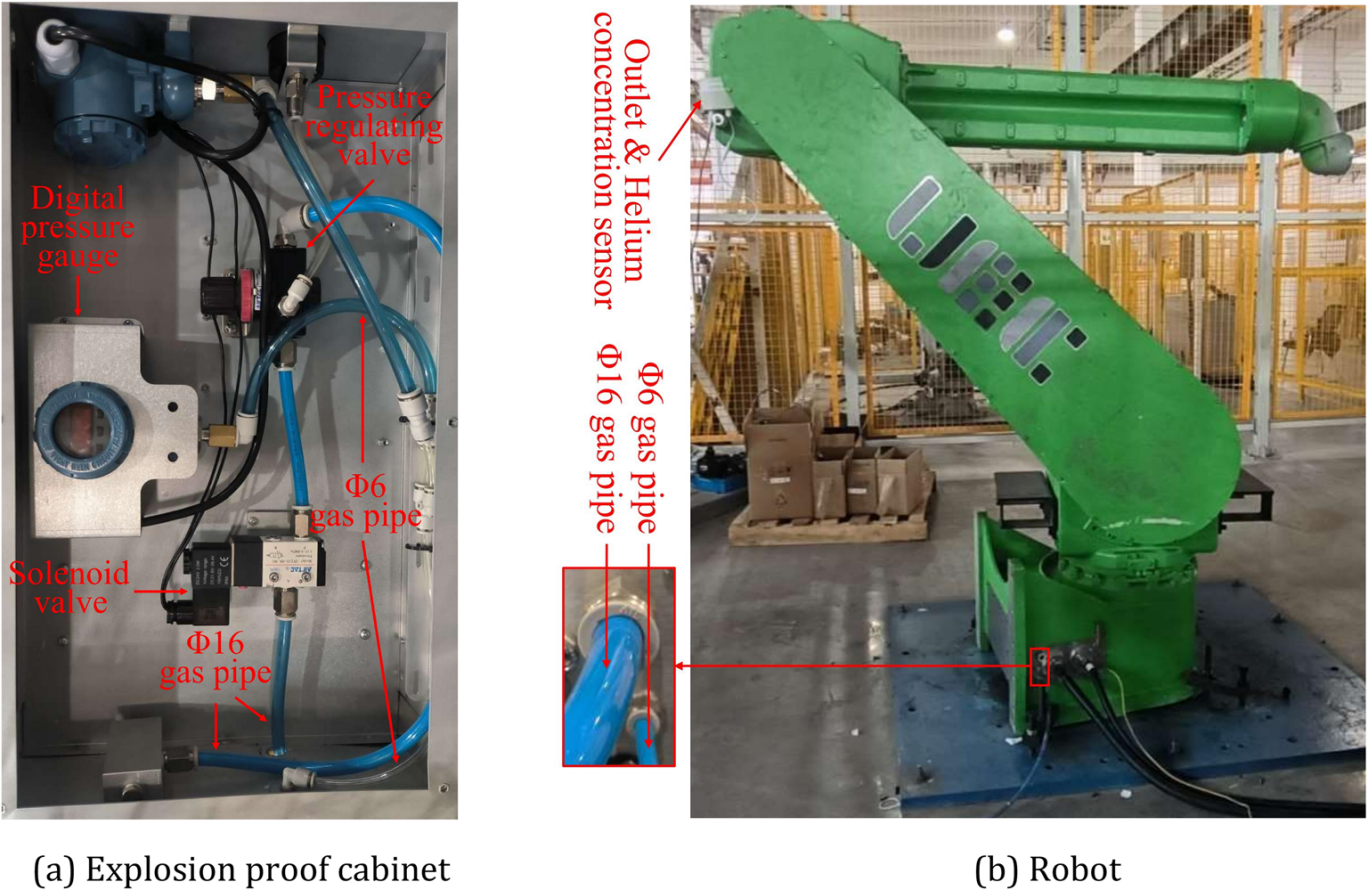
Physical diagram of the positive pressure explosion-proof robot experimental platform. (**a**) Explosion proof cabinet, (**b**) robot.

To verify the accuracy of the simulation results, a positive-pressure explosion-proof robot experimental platform was designed and built, and the schematic and physical diagrams are shown in Figs. 18 and 19. The experiment platform mainly includes control cabinet, explosion-proof cabinet, robot body, air compressor, helium cylinder, helium concentration sensor, gas pipe and so on. The Φ16 gas pipe is used to provide pure air and the Φ6 gas pipe is used to measure the pressure inside the robot positive pressure cavity. The helium concentration sensor probe is installed at the robot outlet at the beginning of the experiment, helium is filled into the cavity from the inlet of the robot. When the helium concentration sensor shows a concentration value greater than 75%, the helium is quickly stopped. Then use the air compressor to provide pure air for ventilation experiments, according to the pre-designed six air intake methods, control and adjust the solenoid valve in the explosion-proof cabinet, regulators, etc. to change the cycle and amplitude parameters of the air intake, when the helium concentration sensor shows that the concentration value is less than 1%, the experiment is completed. The changes in pressure and helium concentration values in the positive pressure cavity are recorded in real time during the experiment.

### Experimental results

**Figure 20:**
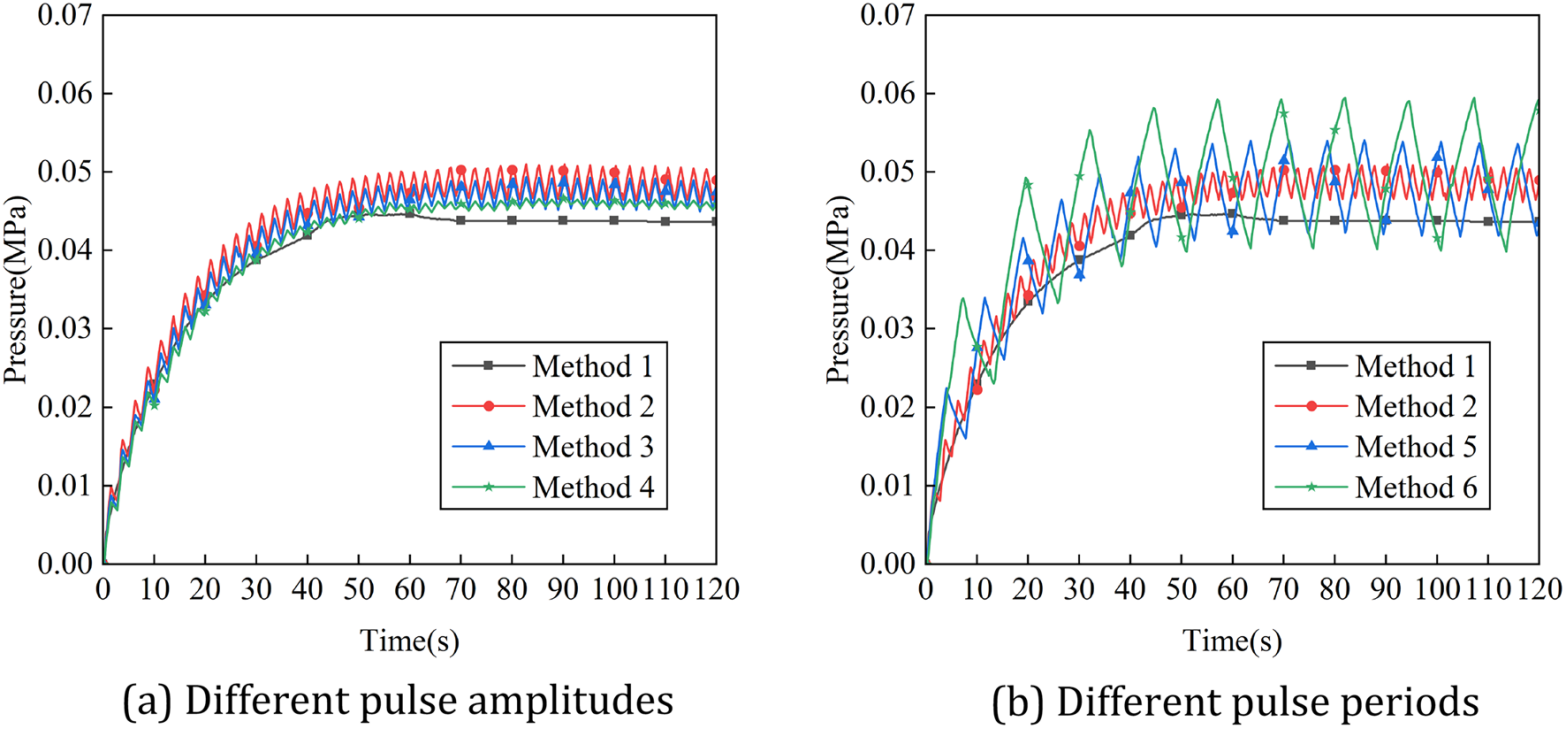
Variation of experimental values of pressure in the positive pressure cavity with time for different intake methods. (**a**) Different pulse amplitudes, (**b**) different pulse periods.

**Figure 21:**
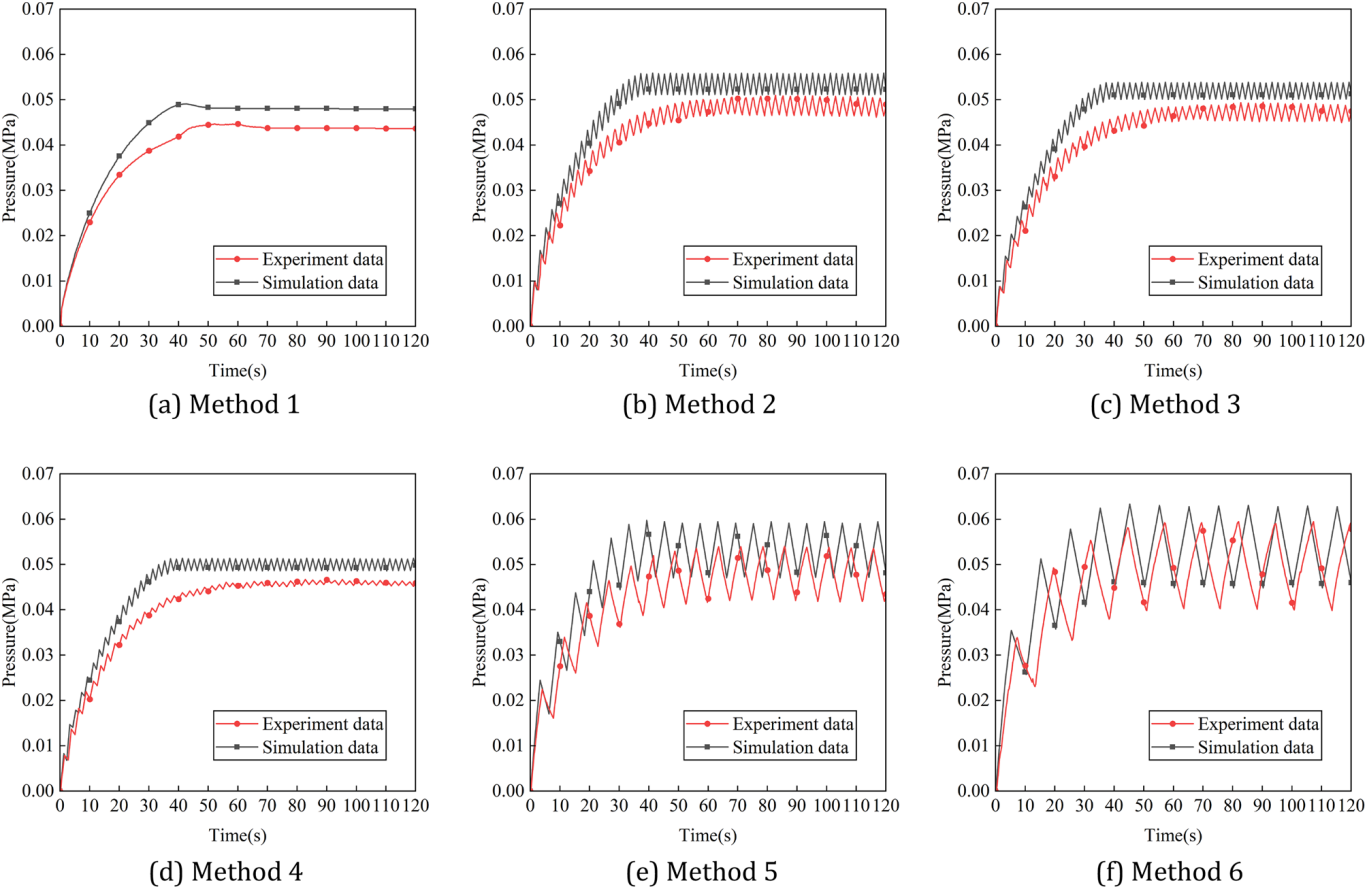
Simulation and experimental comparison of the variation of experimental values of pressure in the positive pressure cavity with time for different intake methods. (**a**) Method 1, (**b**) method 2, (**c**) method 3, (**d**) method 4, (**e**) method 5, (**f**) method 6.

**Figure 22:**
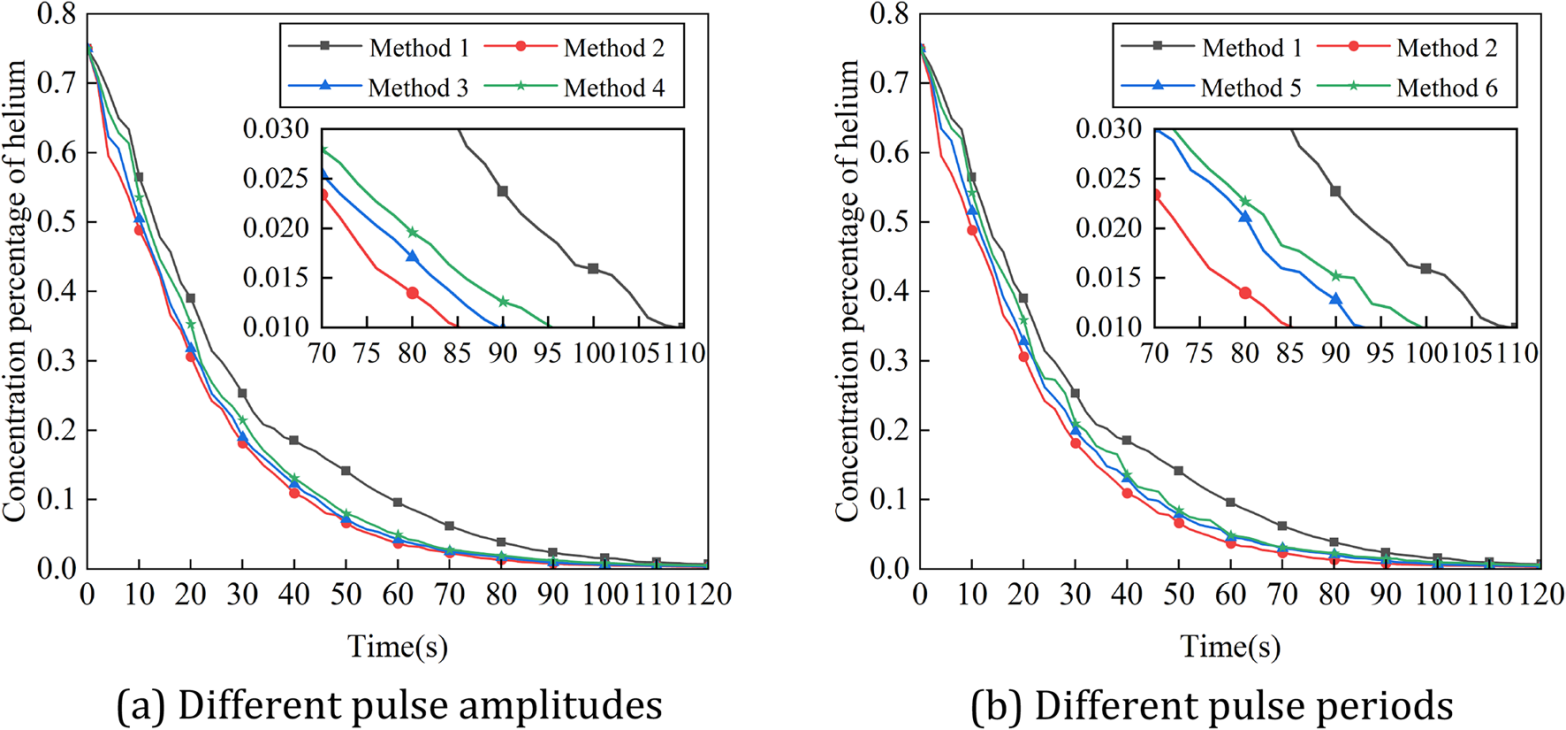
Variation of experimental values of helium concentration with time for different intake methods. (**a**) Different pulse amplitudes. (**b**) Different pulse periods.

**Figure 23:**
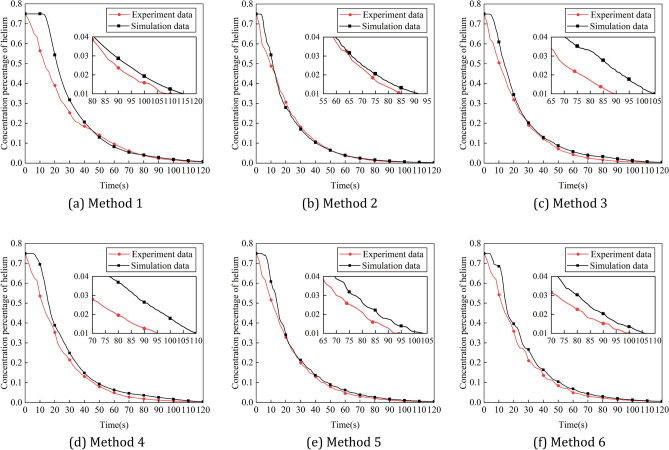
Simulation and experimental comparison of the Variation of experimental values of helium concentration with time for different intake methods. (**a**) Method 1, (**b**) method 2, (**c**) method 3, (**d**) method 4, (**e**) method 5, (**f**) method 6.

Figure 20 shows the variation of experimental values of pressure in the positive pressure cavity with time for different intake methods, and Fig. 21 shows the comparison between the simulation and experimental results. It can be seen that the variation law of pressure in the cavity with pulse amplitude and period is basically consistent with the simulation results. However, the difference between the experimental results and the simulation results of the pressure in the cavity is large. This is due to the poor sealing effect of the robot, and a small amount of gas leakage occur during the ventilation process, resulting in a decrease in the cavity pressure value. Figure 22 shows the variation of experimental values of helium concentration with time for different intake methods, and Fig. 23 shows the comparison between the simulation and experimental results. It can be seen that the pulse intake is beneficial to improve the ventilation efficiency of the explosion-proof robot, and the effect law of pulse amplitude and period on the ventilation efficiency is consistent with the simulation results. The trends of the experimental and simulation results under six different intake methods basically match, which proves the accuracy of the ventilation simulation results.

## Conclusions

In this study, the ventilation method inside the positive pressure cavity of an explosion-proof robot is investigated using a positive pressure explosion-proof robot as an example. Focused on the difference of ventilation efficiency under constant pressure intake and pulse intake methods, analyzed the effect of the amplitude and period of pulse intake on ventilation efficiency, and provided reference for the design of ventilation system for positive pressure explosion-proof robots, the main conclusions are as follows:

Compared with the constant pressure air intake, pulse intake can improve the ventilation efficiency, the reason is that pulse intake causes large fluctuations in the flow field inside the robot positive pressure explosion-proof cavity, the mixing effect of pure air and explosive gases inside the cavity is increasing, thus accelerating the diffusion of explosive gases and effectively avoiding the accumulation of gases.

For pulse intake methods with the same period, the greater the amplitude the higher the ventilation efficiency, because the greater the amplitude of the pulse intake, the better it can drive the flow of air throughout the cavity, and the higher the ventilation efficiency of the positive pressure explosion-proof robot.

For pulse intake methods with the same amplitude, the smaller the period the higher the ventilation efficiency, because the smaller the period of the pulse intake, the more times the airflow is disturbed in the cavity, and therefore the higher the ventilation efficiency (Supplementary Information S1).

### Supplementary Information

Supplementary Information.

## Data Availability

The datasets used and/or analyzed during the current study are available from the corresponding author on reasonable request.
