# Supplementary material for: Influence of periodic pulse intake on the ventilation efficiency of positive pressure explosion-proof robot
Source: Sci Rep. 2024 Jan 16;14:1433. doi: 10.1038/s41598-024-52011-9 (PMC10791746; doi:10.1038/s41598-024-52011-9)
Supplement: Supplementary file 1 — Supplementary Information. [file 41598_2024_52011_MOESM1_ESM.docx]

| Variation of experimental values of helium concentration with time for different intake methods | | | | | | |
| --- | --- | --- | --- | --- | --- | --- |
| Time(s) | Method1 | Method2 | Method3 | Method4 | Method5 | Method6 |
| 0 | 0.75 | 0.75 | 0.75 | 0.75 | 0.75 | 0.75 |
| 2 | 0.7252 | 0.7003 | 0.7053 | 0.7094 | 0.7126 | 0.7164 |
| 4 | 0.6905 | 0.596 | 0.6235 | 0.6602 | 0.6351 | 0.6671 |
| 6 | 0.6502 | 0.57 | 0.6062 | 0.6289 | 0.6176 | 0.6357 |
| 8 | 0.6337 | 0.5348 | 0.5525 | 0.6137 | 0.5637 | 0.6204 |
| 10 | 0.5648 | 0.4886 | 0.5052 | 0.5361 | 0.5162 | 0.5427 |
| 12 | 0.5248 | 0.4588 | 0.465 | 0.4886 | 0.4758 | 0.4951 |
| 14 | 0.4785 | 0.4218 | 0.4286 | 0.4466 | 0.4392 | 0.453 |
| 16 | 0.4573 | 0.3659 | 0.3816 | 0.4191 | 0.392 | 0.4254 |
| 18 | 0.4132 | 0.3448 | 0.3525 | 0.391 | 0.3627 | 0.3972 |
| 20 | 0.3902 | 0.3061 | 0.318 | 0.353 | 0.328 | 0.3591 |
| 22 | 0.3537 | 0.2727 | 0.2906 | 0.2974 | 0.3004 | 0.3034 |
| 24 | 0.3148 | 0.2427 | 0.253 | 0.2692 | 0.2626 | 0.2751 |
| 26 | 0.2985 | 0.2307 | 0.2371 | 0.2483 | 0.2465 | 0.2731 |
| 28 | 0.2773 | 0.2034 | 0.2195 | 0.2349 | 0.2287 | 0.2536 |
| 30 | 0.2532 | 0.1816 | 0.1902 | 0.2145 | 0.1992 | 0.2101 |
| 32 | 0.2268 | 0.1664 | 0.1734 | 0.1907 | 0.1822 | 0.1992 |
| 34 | 0.2087 | 0.15 | 0.1611 | 0.1715 | 0.1697 | 0.1779 |
| 36 | 0.2028 | 0.1377 | 0.1481 | 0.1579 | 0.1485 | 0.1702 |
| 38 | 0.1902 | 0.1243 | 0.1349 | 0.1426 | 0.1431 | 0.1658 |
| 40 | 0.1855 | 0.1096 | 0.1228 | 0.1311 | 0.1308 | 0.1362 |
| 42 | 0.1764 | 0.1024 | 0.1106 | 0.1209 | 0.1134 | 0.1189 |
| 44 | 0.1705 | 0.0922 | 0.1033 | 0.1101 | 0.1009 | 0.115 |
| 46 | 0.1595 | 0.0809 | 0.091 | 0.1007 | 0.0984 | 0.1118 |
| 48 | 0.1504 | 0.078 | 0.0799 | 0.0893 | 0.0871 | 0.094 |
| 50 | 0.1413 | 0.0666 | 0.0722 | 0.0802 | 0.0792 | 0.0848 |
| 52 | 0.1308 | 0.0577 | 0.0639 | 0.075 | 0.0707 | 0.0755 |
| 54 | 0.1206 | 0.0524 | 0.0573 | 0.0673 | 0.0639 | 0.0717 |
| 56 | 0.1117 | 0.0476 | 0.0542 | 0.0613 | 0.0606 | 0.0706 |
| 58 | 0.104 | 0.0419 | 0.0483 | 0.0538 | 0.0575 | 0.06 |
| 60 | 0.0958 | 0.0372 | 0.0428 | 0.0499 | 0.0458 | 0.049 |
| 62 | 0.0885 | 0.0332 | 0.0386 | 0.0429 | 0.0444 | 0.0469 |
| 64 | 0.0823 | 0.0326 | 0.0354 | 0.0403 | 0.041 | 0.0442 |
| 66 | 0.076 | 0.0278 | 0.0327 | 0.0351 | 0.0361 | 0.0389 |
| 68 | 0.0682 | 0.0257 | 0.028 | 0.0307 | 0.0342 | 0.0344 |
| 70 | 0.062 | 0.0234 | 0.0255 | 0.028 | 0.0301 | 0.0316 |
| 72 | 0.057 | 0.0211 | 0.0235 | 0.0266 | 0.0289 | 0.0301 |
| 74 | 0.0516 | 0.0185 | 0.0219 | 0.0245 | 0.0259 | 0.0279 |
| 76 | 0.0468 | 0.016 | 0.0203 | 0.0227 | 0.0247 | 0.026 |
| 78 | 0.0433 | 0.0148 | 0.0189 | 0.0213 | 0.0231 | 0.0245 |
| 80 | 0.0386 | 0.0135 | 0.0171 | 0.0196 | 0.0211 | 0.0227 |
| 82 | 0.0356 | 0.0122 | 0.0153 | 0.0184 | 0.0178 | 0.0214 |
| 84 | 0.0321 | 0.0105 | 0.0138 | 0.0164 | 0.016 | 0.0183 |
| 86 | 0.0283 | 0.0097 | 0.0122 | 0.0149 | 0.0156 | 0.0177 |
| 88 | 0.0265 | 0.0087 | 0.0108 | 0.0137 | 0.014 | 0.0164 |
| 90 | 0.0237 | 0.0083 | 0.0098 | 0.0126 | 0.0128 | 0.0152 |
| 92 | 0.0215 | 0.0073 | 0.0089 | 0.012 | 0.0103 | 0.015 |
| 94 | 0.0199 | 0.007 | 0.008 | 0.0108 | 0.0098 | 0.0124 |
| 96 | 0.0185 | 0.0064 | 0.0076 | 0.0097 | 0.0088 | 0.012 |
| 98 | 0.0163 | 0.0062 | 0.0071 | 0.0093 | 0.0079 | 0.0107 |
| 100 | 0.0159 | 0.0059 | 0.0068 | 0.0089 | 0.0071 | 0.0098 |
| 102 | 0.0153 | 0.0052 | 0.0065 | 0.0084 | 0.0067 | 0.0094 |
| 104 | 0.0135 | 0.005 | 0.0061 | 0.0079 | 0.0063 | 0.0092 |
| 106 | 0.011 | 0.0049 | 0.0057 | 0.0074 | 0.006 | 0.0086 |
| 108 | 0.0102 | 0.0046 | 0.0053 | 0.007 | 0.0058 | 0.0082 |
| 110 | 0.0099 | 0.0045 | 0.005 | 0.0068 | 0.0056 | 0.0076 |
| 112 | 0.0091 | 0.0042 | 0.0048 | 0.0065 | 0.0053 | 0.0073 |
| 114 | 0.0085 | 0.004 | 0.0047 | 0.0061 | 0.005 | 0.0068 |
| 116 | 0.008 | 0.0039 | 0.0044 | 0.0058 | 0.0047 | 0.0064 |
| 118 | 0.0076 | 0.0036 | 0.0041 | 0.0055 | 0.0045 | 0.0062 |
| 120 | 0.0071 | 0.0035 | 0.0039 | 0.0052 | 0.0042 | 0.0059 |

| Variation of experimental values of pressure in the positive pressure cavity with time for different intake methods (Mpa) | | | | | | |
| --- | --- | --- | --- | --- | --- | --- |
| Time(s) | Method1 | Method2 | Method3 | Method4 | Method5 | Method6 |
| 0 | 0 | 0 | 0 | 0 | 0 | 0 |
| 0.25 | 0.0005 | 0.0008 | 0.0007 | 0.0008 | 0.0009 | 0.0004 |
| 0.5 | 0.0039 | 0.0029 | 0.0028 | 0.0026 | 0.0027 | 0.0014 |
| 0.75 | 0.0046 | 0.0049 | 0.0044 | 0.0039 | 0.0046 | 0.0032 |
| 1 | 0.0054 | 0.0068 | 0.0061 | 0.0052 | 0.0063 | 0.0051 |
| 1.25 | 0.0062 | 0.0086 | 0.0077 | 0.0066 | 0.0081 | 0.0069 |
| 1.5 | 0.0068 | 0.0099 | 0.0088 | 0.0079 | 0.0093 | 0.0082 |
| 1.75 | 0.0075 | 0.0095 | 0.0085 | 0.0077 | 0.0109 | 0.0089 |
| 2 | 0.0081 | 0.0088 | 0.0079 | 0.0075 | 0.0125 | 0.0099 |
| 2.25 | 0.0089 | 0.0087 | 0.0078 | 0.0073 | 0.0139 | 0.0115 |
| 2.5 | 0.0095 | 0.0083 | 0.0075 | 0.0071 | 0.0151 | 0.0131 |
| 2.75 | 0.0101 | 0.0081 | 0.0074 | 0.0069 | 0.0165 | 0.0142 |
| 3 | 0.0105 | 0.0098 | 0.0091 | 0.0086 | 0.0179 | 0.0157 |
| 3.25 | 0.0111 | 0.0114 | 0.0105 | 0.0103 | 0.0192 | 0.0171 |
| 3.5 | 0.0117 | 0.0147 | 0.0136 | 0.0121 | 0.0202 | 0.0185 |
| 3.75 | 0.0122 | 0.0159 | 0.0146 | 0.0137 | 0.0216 | 0.0196 |
| 4 | 0.0128 | 0.0155 | 0.0143 | 0.0135 | 0.0225 | 0.0211 |
| 4.25 | 0.0132 | 0.0148 | 0.0137 | 0.0132 | 0.0217 | 0.0223 |
| 4.5 | 0.0138 | 0.0144 | 0.0134 | 0.0129 | 0.0214 | 0.0226 |
| 4.75 | 0.0143 | 0.0141 | 0.0131 | 0.0127 | 0.0207 | 0.0236 |
| 5 | 0.0149 | 0.0138 | 0.0129 | 0.0125 | 0.0203 | 0.0246 |
| 5.25 | 0.0152 | 0.0154 | 0.0144 | 0.0137 | 0.0198 | 0.0259 |
| 5.5 | 0.0158 | 0.0167 | 0.0155 | 0.0148 | 0.0195 | 0.0271 |
| 5.75 | 0.0164 | 0.0183 | 0.0169 | 0.0159 | 0.0191 | 0.0284 |
| 6 | 0.0167 | 0.0198 | 0.0182 | 0.0171 | 0.0186 | 0.0293 |
| 6.25 | 0.0173 | 0.0209 | 0.0191 | 0.0183 | 0.0182 | 0.0306 |
| 6.5 | 0.0175 | 0.0205 | 0.0188 | 0.0181 | 0.0179 | 0.0316 |
| 6.75 | 0.0181 | 0.0197 | 0.0187 | 0.0178 | 0.0175 | 0.0328 |
| 7 | 0.0184 | 0.0193 | 0.0184 | 0.0176 | 0.0171 | 0.0337 |
| 7.25 | 0.0188 | 0.0188 | 0.018 | 0.0173 | 0.0168 | 0.0339 |
| 7.5 | 0.0192 | 0.0184 | 0.0177 | 0.0171 | 0.0164 | 0.0334 |
| 7.75 | 0.0196 | 0.0201 | 0.0191 | 0.0181 | 0.0161 | 0.0327 |
| 8 | 0.0201 | 0.0212 | 0.0201 | 0.0191 | 0.0176 | 0.0315 |
| 8.25 | 0.0205 | 0.0227 | 0.0214 | 0.0201 | 0.0192 | 0.0309 |
| 8.5 | 0.0209 | 0.0241 | 0.0226 | 0.021 | 0.0203 | 0.0304 |
| 8.75 | 0.0212 | 0.0251 | 0.0234 | 0.0221 | 0.0217 | 0.0299 |
| 9 | 0.0216 | 0.0247 | 0.0231 | 0.0217 | 0.0231 | 0.0294 |
| 9.25 | 0.0221 | 0.0239 | 0.0225 | 0.0213 | 0.0244 | 0.0289 |
| 9.5 | 0.0224 | 0.0232 | 0.0218 | 0.0209 | 0.0253 | 0.0285 |
| 9.75 | 0.0226 | 0.0228 | 0.0215 | 0.0206 | 0.0267 | 0.0281 |
| 10 | 0.023 | 0.0223 | 0.0211 | 0.0203 | 0.0276 | 0.0277 |
| 10.25 | 0.0234 | 0.0236 | 0.0223 | 0.0211 | 0.0289 | 0.0273 |
| 10.5 | 0.0237 | 0.0251 | 0.0234 | 0.0219 | 0.0299 | 0.0268 |
| 10.75 | 0.0241 | 0.0262 | 0.0246 | 0.0227 | 0.0311 | 0.0264 |
| 11 | 0.0244 | 0.0275 | 0.0257 | 0.0235 | 0.0321 | 0.0261 |
| 11.25 | 0.0247 | 0.0285 | 0.0269 | 0.0243 | 0.0332 | 0.0256 |
| 11.5 | 0.0251 | 0.0282 | 0.0264 | 0.0241 | 0.0339 | 0.0252 |
| 11.75 | 0.0253 | 0.0274 | 0.0259 | 0.0239 | 0.0332 | 0.0249 |
| 12 | 0.0257 | 0.0268 | 0.0253 | 0.0237 | 0.0329 | 0.0245 |
| 12.25 | 0.0259 | 0.0263 | 0.0248 | 0.0235 | 0.0321 | 0.0241 |
| 12.5 | 0.0263 | 0.0255 | 0.0243 | 0.0233 | 0.0314 | 0.0248 |
| 12.75 | 0.0266 | 0.0267 | 0.0254 | 0.0242 | 0.0309 | 0.0238 |
| 13 | 0.0269 | 0.0282 | 0.0266 | 0.0251 | 0.0304 | 0.0234 |
| 13.25 | 0.0271 | 0.0295 | 0.0278 | 0.0259 | 0.0299 | 0.0231 |
| 13.5 | 0.0274 | 0.0308 | 0.0289 | 0.0268 | 0.0293 | 0.0236 |
| 13.75 | 0.0277 | 0.0316 | 0.0301 | 0.0277 | 0.0289 | 0.0253 |
| 14 | 0.0281 | 0.0305 | 0.0294 | 0.0274 | 0.0284 | 0.0265 |
| 14.25 | 0.0283 | 0.0298 | 0.0288 | 0.0272 | 0.0277 | 0.028 |
| 14.5 | 0.0285 | 0.0293 | 0.0281 | 0.0269 | 0.0275 | 0.0295 |
| 14.75 | 0.0287 | 0.0286 | 0.0274 | 0.0266 | 0.0268 | 0.0309 |
| 15 | 0.029 | 0.0296 | 0.0284 | 0.0273 | 0.0266 | 0.0319 |
| 15.25 | 0.0293 | 0.031 | 0.0295 | 0.0281 | 0.0261 | 0.0334 |
| 15.5 | 0.0295 | 0.0323 | 0.0307 | 0.0288 | 0.0271 | 0.0343 |
| 15.75 | 0.0297 | 0.0336 | 0.0318 | 0.0295 | 0.0286 | 0.0357 |
| 16 | 0.03 | 0.0345 | 0.0329 | 0.0303 | 0.0299 | 0.0366 |
| 16.25 | 0.0303 | 0.034 | 0.0323 | 0.0299 | 0.0311 | 0.0378 |
| 16.5 | 0.0306 | 0.0332 | 0.0317 | 0.0295 | 0.0323 | 0.0387 |
| 16.75 | 0.0307 | 0.0324 | 0.0312 | 0.0291 | 0.0332 | 0.0398 |
| 17 | 0.0309 | 0.0316 | 0.0316 | 0.0287 | 0.0345 | 0.0406 |
| 17.25 | 0.0312 | 0.0312 | 0.0301 | 0.0293 | 0.0353 | 0.0417 |
| 17.5 | 0.0314 | 0.0321 | 0.0309 | 0.0299 | 0.0365 | 0.0426 |
| 17.75 | 0.0316 | 0.0335 | 0.0319 | 0.0306 | 0.0373 | 0.0435 |
| 18 | 0.0318 | 0.0345 | 0.0331 | 0.0313 | 0.0384 | 0.0444 |
| 18.25 | 0.0321 | 0.0358 | 0.0342 | 0.0319 | 0.0393 | 0.0453 |
| 18.5 | 0.0323 | 0.0367 | 0.0352 | 0.0326 | 0.0401 | 0.0458 |
| 18.75 | 0.0325 | 0.0363 | 0.0346 | 0.0324 | 0.0411 | 0.0467 |
| 19 | 0.0326 | 0.0353 | 0.0339 | 0.0322 | 0.0416 | 0.0476 |
| 19.25 | 0.0328 | 0.0349 | 0.0333 | 0.0321 | 0.0409 | 0.0484 |
| 19.5 | 0.0331 | 0.0341 | 0.0327 | 0.0319 | 0.0403 | 0.0493 |
| 19.75 | 0.0333 | 0.0333 | 0.0321 | 0.0317 | 0.0395 | 0.0491 |
| 20 | 0.0335 | 0.0343 | 0.0331 | 0.0323 | 0.0387 | 0.0484 |
| 20.25 | 0.0336 | 0.0356 | 0.0342 | 0.0329 | 0.0379 | 0.0473 |
| 20.5 | 0.0338 | 0.0366 | 0.0352 | 0.0334 | 0.0374 | 0.0466 |
| 20.75 | 0.0339 | 0.0379 | 0.0363 | 0.0339 | 0.0366 | 0.0458 |
| 21 | 0.0342 | 0.0388 | 0.0372 | 0.0346 | 0.0361 | 0.0451 |
| 21.25 | 0.0342 | 0.0384 | 0.0366 | 0.0344 | 0.0355 | 0.0442 |
| 21.5 | 0.0344 | 0.0374 | 0.0361 | 0.0342 | 0.0349 | 0.0436 |
| 21.75 | 0.0346 | 0.0366 | 0.0355 | 0.0339 | 0.0343 | 0.0427 |
| 22 | 0.0348 | 0.0359 | 0.0349 | 0.0338 | 0.0337 | 0.0422 |
| 22.25 | 0.035 | 0.0355 | 0.0343 | 0.0336 | 0.0331 | 0.0413 |
| 22.5 | 0.0351 | 0.0362 | 0.0353 | 0.0342 | 0.0326 | 0.0408 |
| 22.75 | 0.0352 | 0.0373 | 0.0362 | 0.0348 | 0.0321 | 0.0402 |
| 23 | 0.0354 | 0.0386 | 0.0373 | 0.0354 | 0.0329 | 0.0396 |
| 23.25 | 0.0356 | 0.0395 | 0.0383 | 0.0359 | 0.0344 | 0.0391 |
| 23.5 | 0.0356 | 0.0407 | 0.0392 | 0.0366 | 0.0354 | 0.0384 |
| 23.75 | 0.0358 | 0.0402 | 0.0386 | 0.0364 | 0.0367 | 0.0376 |
| 24 | 0.0361 | 0.0392 | 0.0379 | 0.0362 | 0.0376 | 0.0369 |
| 24.25 | 0.0361 | 0.0384 | 0.0373 | 0.0361 | 0.0388 | 0.0365 |
| 24.5 | 0.0363 | 0.0376 | 0.0367 | 0.0359 | 0.0396 | 0.0359 |
| 24.75 | 0.0363 | 0.0372 | 0.0361 | 0.0357 | 0.0407 | 0.0353 |
| 25 | 0.0365 | 0.0377 | 0.0369 | 0.0362 | 0.0415 | 0.0351 |
| 25.25 | 0.0366 | 0.0392 | 0.0378 | 0.0366 | 0.0424 | 0.0344 |
| 25.5 | 0.0368 | 0.0401 | 0.0387 | 0.0371 | 0.0433 | 0.0339 |
| 25.75 | 0.0369 | 0.0413 | 0.0397 | 0.0375 | 0.0442 | 0.0333 |
| 26 | 0.037 | 0.0421 | 0.0405 | 0.0381 | 0.0451 | 0.0336 |
| 26.25 | 0.0371 | 0.0418 | 0.0399 | 0.0378 | 0.0459 | 0.0349 |
| 26.5 | 0.0373 | 0.0407 | 0.0392 | 0.0376 | 0.0465 | 0.0362 |
| 26.75 | 0.0374 | 0.0399 | 0.0396 | 0.0373 | 0.0459 | 0.0375 |
| 27 | 0.0375 | 0.0395 | 0.0391 | 0.0371 | 0.0448 | 0.0383 |
| 27.25 | 0.0376 | 0.0387 | 0.0375 | 0.0369 | 0.0441 | 0.0396 |
| 27.5 | 0.0377 | 0.0393 | 0.0383 | 0.0374 | 0.0432 | 0.0405 |
| 27.75 | 0.0379 | 0.0407 | 0.0392 | 0.0379 | 0.0424 | 0.0417 |
| 28 | 0.038 | 0.0415 | 0.0401 | 0.0385 | 0.0419 | 0.0426 |
| 28.25 | 0.038 | 0.0428 | 0.0411 | 0.0389 | 0.0411 | 0.0437 |
| 28.5 | 0.0382 | 0.0435 | 0.0419 | 0.0395 | 0.0405 | 0.0445 |
| 28.75 | 0.0383 | 0.0432 | 0.0413 | 0.0393 | 0.0397 | 0.0454 |
| 29 | 0.0384 | 0.0421 | 0.0408 | 0.0391 | 0.0391 | 0.0463 |
| 29.25 | 0.0384 | 0.0413 | 0.0402 | 0.0388 | 0.0385 | 0.0472 |
| 29.5 | 0.0386 | 0.0409 | 0.0395 | 0.0386 | 0.0381 | 0.0481 |
| 29.75 | 0.0387 | 0.0401 | 0.0389 | 0.0384 | 0.0374 | 0.0489 |
| 30 | 0.0388 | 0.0406 | 0.0397 | 0.0388 | 0.0369 | 0.0495 |
| 30.25 | 0.0389 | 0.0419 | 0.0405 | 0.0392 | 0.0362 | 0.0503 |
| 30.5 | 0.0389 | 0.0428 | 0.0414 | 0.0396 | 0.0371 | 0.0512 |
| 30.75 | 0.039 | 0.0441 | 0.0422 | 0.0401 | 0.0385 | 0.0518 |
| 31 | 0.0391 | 0.0447 | 0.0431 | 0.0404 | 0.0394 | 0.0526 |
| 31.25 | 0.0392 | 0.0443 | 0.0425 | 0.0402 | 0.0407 | 0.0532 |
| 31.5 | 0.0392 | 0.0433 | 0.0418 | 0.0401 | 0.0415 | 0.0541 |
| 31.75 | 0.0393 | 0.0426 | 0.0412 | 0.0399 | 0.0426 | 0.0546 |
| 32 | 0.0394 | 0.0418 | 0.0405 | 0.0397 | 0.0434 | 0.0554 |
| 32.25 | 0.0395 | 0.041 | 0.0399 | 0.0395 | 0.0443 | 0.0551 |
| 32.5 | 0.0396 | 0.0418 | 0.0407 | 0.0399 | 0.0452 | 0.0545 |
| 32.75 | 0.0396 | 0.0429 | 0.0416 | 0.0403 | 0.0461 | 0.0533 |
| 33 | 0.0397 | 0.0437 | 0.0424 | 0.0407 | 0.0469 | 0.0526 |
| 33.25 | 0.0398 | 0.0449 | 0.0432 | 0.0411 | 0.0477 | 0.0516 |
| 33.5 | 0.0399 | 0.0457 | 0.0441 | 0.0415 | 0.0483 | 0.0508 |
| 33.75 | 0.0399 | 0.0453 | 0.0435 | 0.0413 | 0.0492 | 0.0499 |
| 34 | 0.04 | 0.0445 | 0.0429 | 0.0411 | 0.0497 | 0.0491 |
| 34.25 | 0.0401 | 0.0438 | 0.0422 | 0.0409 | 0.0488 | 0.0483 |
| 34.5 | 0.0402 | 0.043 | 0.0417 | 0.0408 | 0.0481 | 0.0477 |
| 34.75 | 0.0403 | 0.0422 | 0.041 | 0.0406 | 0.047 | 0.0468 |
| 35 | 0.0403 | 0.0426 | 0.0418 | 0.041 | 0.0463 | 0.0463 |
| 35.25 | 0.0404 | 0.0436 | 0.0427 | 0.0414 | 0.0454 | 0.0454 |
| 35.5 | 0.0405 | 0.0447 | 0.0435 | 0.0418 | 0.0449 | 0.0449 |
| 35.75 | 0.0406 | 0.0455 | 0.0443 | 0.0422 | 0.0441 | 0.0443 |
| 36 | 0.0407 | 0.0466 | 0.0451 | 0.0426 | 0.0433 | 0.0434 |
| 36.25 | 0.0407 | 0.0462 | 0.0444 | 0.0424 | 0.0427 | 0.0429 |
| 36.5 | 0.0408 | 0.0451 | 0.0436 | 0.0422 | 0.0417 | 0.0423 |
| 36.75 | 0.0409 | 0.0445 | 0.0429 | 0.042 | 0.0419 | 0.0417 |
| 37 | 0.041 | 0.0436 | 0.0421 | 0.0418 | 0.0413 | 0.0412 |
| 37.25 | 0.041 | 0.0428 | 0.0416 | 0.0416 | 0.0408 | 0.0403 |
| 37.5 | 0.0411 | 0.0435 | 0.0424 | 0.0419 | 0.0399 | 0.0398 |
| 37.75 | 0.0412 | 0.0446 | 0.0433 | 0.0422 | 0.0394 | 0.0392 |
| 38 | 0.0413 | 0.0454 | 0.0441 | 0.0425 | 0.0388 | 0.0386 |
| 38.25 | 0.0414 | 0.0465 | 0.0449 | 0.0428 | 0.0396 | 0.038 |
| 38.5 | 0.0414 | 0.0473 | 0.0457 | 0.0431 | 0.0408 | 0.0383 |
| 38.75 | 0.0415 | 0.0469 | 0.0451 | 0.0429 | 0.042 | 0.0395 |
| 39 | 0.0416 | 0.0461 | 0.0445 | 0.0427 | 0.0429 | 0.0408 |
| 39.25 | 0.0417 | 0.0451 | 0.0438 | 0.0425 | 0.044 | 0.0419 |
| 39.5 | 0.0417 | 0.0446 | 0.0432 | 0.0423 | 0.0456 | 0.0428 |
| 39.75 | 0.0418 | 0.0438 | 0.0426 | 0.0421 | 0.0465 | 0.0441 |
| 40 | 0.0419 | 0.0448 | 0.0432 | 0.0424 | 0.0474 | 0.0449 |
| 40.25 | 0.042 | 0.0442 | 0.0439 | 0.0427 | 0.0482 | 0.046 |
| 40.5 | 0.0422 | 0.045 | 0.0445 | 0.0429 | 0.0491 | 0.0468 |
| 40.75 | 0.0423 | 0.0462 | 0.0451 | 0.0432 | 0.0497 | 0.0477 |
| 41 | 0.0424 | 0.0469 | 0.0458 | 0.0435 | 0.0506 | 0.0486 |
| 41.25 | 0.0426 | 0.048 | 0.0464 | 0.0438 | 0.0511 | 0.0494 |
| 41.5 | 0.0427 | 0.0476 | 0.0458 | 0.0436 | 0.052 | 0.0502 |
| 41.75 | 0.0428 | 0.0468 | 0.0451 | 0.0434 | 0.0501 | 0.0511 |
| 42 | 0.043 | 0.0458 | 0.0445 | 0.0433 | 0.049 | 0.0517 |
| 42.25 | 0.0431 | 0.0451 | 0.0438 | 0.0431 | 0.0483 | 0.0526 |
| 42.5 | 0.0432 | 0.0442 | 0.0431 | 0.0429 | 0.0475 | 0.0531 |
| 42.75 | 0.0434 | 0.0447 | 0.0438 | 0.0432 | 0.0467 | 0.054 |
| 43 | 0.0435 | 0.0458 | 0.0446 | 0.0434 | 0.0459 | 0.0545 |
| 43.25 | 0.0436 | 0.0466 | 0.0453 | 0.0437 | 0.0453 | 0.0551 |
| 43.5 | 0.0437 | 0.0477 | 0.0461 | 0.0439 | 0.0444 | 0.056 |
| 43.75 | 0.0438 | 0.0484 | 0.0468 | 0.0442 | 0.0439 | 0.0565 |
| 44 | 0.0438 | 0.048 | 0.0461 | 0.0439 | 0.043 | 0.0571 |
| 44.25 | 0.0438 | 0.0472 | 0.0454 | 0.0438 | 0.0425 | 0.0577 |
| 44.5 | 0.044 | 0.0462 | 0.0448 | 0.0435 | 0.0416 | 0.0582 |
| 44.75 | 0.044 | 0.0454 | 0.0441 | 0.0433 | 0.0411 | 0.0581 |
| 45 | 0.044 | 0.0446 | 0.0434 | 0.0431 | 0.0405 | 0.0574 |
| 45.25 | 0.044 | 0.0451 | 0.0441 | 0.0434 | 0.0412 | 0.0562 |
| 45.5 | 0.044 | 0.0463 | 0.0449 | 0.0437 | 0.0423 | 0.0554 |
| 45.75 | 0.0441 | 0.047 | 0.0456 | 0.044 | 0.0436 | 0.0543 |
| 46 | 0.0442 | 0.0481 | 0.0464 | 0.0443 | 0.0444 | 0.0535 |
| 46.25 | 0.0442 | 0.0488 | 0.0472 | 0.0446 | 0.0455 | 0.0524 |
| 46.5 | 0.0442 | 0.0485 | 0.0466 | 0.0443 | 0.0463 | 0.0519 |
| 46.75 | 0.0442 | 0.0478 | 0.0459 | 0.044 | 0.0471 | 0.051 |
| 47 | 0.0443 | 0.0467 | 0.0453 | 0.0437 | 0.048 | 0.0502 |
| 47.25 | 0.0443 | 0.046 | 0.0446 | 0.0434 | 0.0488 | 0.0493 |
| 47.5 | 0.0443 | 0.0451 | 0.0439 | 0.0436 | 0.0496 | 0.0486 |
| 47.75 | 0.0443 | 0.0455 | 0.0446 | 0.0439 | 0.0502 | 0.0479 |
| 48 | 0.0443 | 0.0462 | 0.0454 | 0.0442 | 0.0511 | 0.0471 |
| 48.25 | 0.0444 | 0.0474 | 0.0461 | 0.0444 | 0.0516 | 0.0465 |
| 48.5 | 0.0444 | 0.0481 | 0.0469 | 0.0447 | 0.0524 | 0.0457 |
| 48.75 | 0.0444 | 0.0492 | 0.0476 | 0.045 | 0.053 | 0.0451 |
| 49 | 0.0444 | 0.0489 | 0.0471 | 0.0448 | 0.0522 | 0.0443 |
| 49.25 | 0.0444 | 0.0481 | 0.0465 | 0.0446 | 0.0514 | 0.0437 |
| 49.5 | 0.0445 | 0.0471 | 0.0458 | 0.0445 | 0.0503 | 0.0431 |
| 49.75 | 0.0445 | 0.0463 | 0.0451 | 0.0443 | 0.0495 | 0.0423 |
| 50 | 0.0445 | 0.0455 | 0.0443 | 0.0441 | 0.0487 | 0.0417 |
| 50.25 | 0.0445 | 0.0458 | 0.045 | 0.0443 | 0.0479 | 0.0411 |
| 50.5 | 0.0445 | 0.0466 | 0.0458 | 0.0446 | 0.047 | 0.0406 |
| 50.75 | 0.0445 | 0.0477 | 0.0465 | 0.0448 | 0.0462 | 0.04 |
| 51 | 0.0445 | 0.0484 | 0.0472 | 0.0451 | 0.0456 | 0.0399 |
| 51.25 | 0.0446 | 0.0495 | 0.0479 | 0.0453 | 0.0447 | 0.0414 |
| 51.5 | 0.0446 | 0.0492 | 0.0473 | 0.0451 | 0.0441 | 0.0424 |
| 51.75 | 0.0446 | 0.0484 | 0.0467 | 0.0449 | 0.0433 | 0.0436 |
| 52 | 0.0447 | 0.0474 | 0.046 | 0.0448 | 0.0427 | 0.0445 |
| 52.25 | 0.0447 | 0.0467 | 0.0454 | 0.0446 | 0.0419 | 0.0457 |
| 52.5 | 0.0446 | 0.0459 | 0.0448 | 0.0444 | 0.0413 | 0.0465 |
| 52.75 | 0.0446 | 0.0461 | 0.0455 | 0.0446 | 0.0421 | 0.0476 |
| 53 | 0.0445 | 0.0469 | 0.0462 | 0.0449 | 0.0432 | 0.0484 |
| 53.25 | 0.0446 | 0.048 | 0.0468 | 0.0451 | 0.0444 | 0.0493 |
| 53.5 | 0.0446 | 0.0487 | 0.0475 | 0.0454 | 0.0452 | 0.0502 |
| 53.75 | 0.0445 | 0.0498 | 0.0482 | 0.0456 | 0.0463 | 0.0507 |
| 54 | 0.0445 | 0.0495 | 0.0476 | 0.0454 | 0.0479 | 0.0516 |
| 54.25 | 0.0445 | 0.0484 | 0.047 | 0.0452 | 0.0488 | 0.0522 |
| 54.5 | 0.0445 | 0.0478 | 0.0463 | 0.045 | 0.0493 | 0.0531 |
| 54.75 | 0.0445 | 0.047 | 0.0457 | 0.0448 | 0.0502 | 0.0537 |
| 55 | 0.0445 | 0.0463 | 0.0451 | 0.0446 | 0.051 | 0.0544 |
| 55.25 | 0.0445 | 0.0471 | 0.0459 | 0.045 | 0.0516 | 0.0551 |
| 55.5 | 0.0445 | 0.0482 | 0.0467 | 0.0453 | 0.0522 | 0.0556 |
| 55.75 | 0.0446 | 0.0489 | 0.0475 | 0.0457 | 0.053 | 0.0565 |
| 56 | 0.0446 | 0.0499 | 0.0483 | 0.0461 | 0.0536 | 0.057 |
| 56.25 | 0.0445 | 0.0497 | 0.0477 | 0.0459 | 0.0528 | 0.0576 |
| 56.5 | 0.0446 | 0.049 | 0.0471 | 0.0457 | 0.052 | 0.0581 |
| 56.75 | 0.0445 | 0.0479 | 0.0466 | 0.0455 | 0.0509 | 0.0587 |
| 57 | 0.0446 | 0.0473 | 0.046 | 0.0453 | 0.0501 | 0.0593 |
| 57.25 | 0.0446 | 0.0465 | 0.0454 | 0.0451 | 0.049 | 0.0591 |
| 57.5 | 0.0446 | 0.0473 | 0.0462 | 0.0453 | 0.0485 | 0.0583 |
| 57.75 | 0.0446 | 0.0484 | 0.0469 | 0.0455 | 0.0476 | 0.0571 |
| 58 | 0.0446 | 0.0491 | 0.0477 | 0.0457 | 0.0468 | 0.0563 |
| 58.25 | 0.0447 | 0.0501 | 0.0485 | 0.0459 | 0.046 | 0.0551 |
| 58.5 | 0.0446 | 0.0499 | 0.0478 | 0.0457 | 0.0454 | 0.0544 |
| 58.75 | 0.0446 | 0.0492 | 0.0472 | 0.0455 | 0.0445 | 0.0536 |
| 59 | 0.0446 | 0.0481 | 0.0465 | 0.0454 | 0.0439 | 0.0527 |
| 59.25 | 0.0446 | 0.0474 | 0.0459 | 0.0452 | 0.0431 | 0.0518 |
| 59.5 | 0.0447 | 0.0464 | 0.0452 | 0.045 | 0.0426 | 0.051 |
| 59.75 | 0.0447 | 0.0467 | 0.0458 | 0.0452 | 0.0417 | 0.0501 |
| 60 | 0.0447 | 0.0474 | 0.0465 | 0.0453 | 0.0425 | 0.0493 |
| 60.25 | 0.0446 | 0.0485 | 0.0471 | 0.0455 | 0.0436 | 0.0488 |
| 60.5 | 0.0446 | 0.0492 | 0.0478 | 0.0456 | 0.0448 | 0.0479 |
| 60.75 | 0.0446 | 0.05 | 0.0484 | 0.0458 | 0.0456 | 0.0473 |
| 61 | 0.0445 | 0.0493 | 0.0477 | 0.0456 | 0.0467 | 0.0464 |
| 61.25 | 0.0445 | 0.0483 | 0.0469 | 0.0453 | 0.0475 | 0.0458 |
| 61.5 | 0.0444 | 0.0476 | 0.0462 | 0.0451 | 0.0483 | 0.0451 |
| 61.75 | 0.0444 | 0.0465 | 0.0453 | 0.0448 | 0.0492 | 0.0445 |
| 62 | 0.0443 | 0.0468 | 0.0459 | 0.045 | 0.0497 | 0.0439 |
| 62.25 | 0.0443 | 0.0475 | 0.0466 | 0.0453 | 0.0506 | 0.043 |
| 62.5 | 0.0443 | 0.0486 | 0.0472 | 0.0455 | 0.0512 | 0.0425 |
| 62.75 | 0.0442 | 0.0493 | 0.0479 | 0.0458 | 0.052 | 0.0419 |
| 63 | 0.0442 | 0.0501 | 0.0485 | 0.046 | 0.0526 | 0.0413 |
| 63.25 | 0.0442 | 0.0494 | 0.0479 | 0.0458 | 0.0535 | 0.0407 |
| 63.5 | 0.0442 | 0.0484 | 0.0474 | 0.0456 | 0.054 | 0.0403 |
| 63.75 | 0.0442 | 0.0477 | 0.0468 | 0.0453 | 0.0532 | 0.0419 |
| 64 | 0.0441 | 0.047 | 0.0463 | 0.0451 | 0.0523 | 0.043 |
| 64.25 | 0.0441 | 0.0469 | 0.0457 | 0.0449 | 0.0512 | 0.0442 |
| 64.5 | 0.0441 | 0.0476 | 0.0463 | 0.0452 | 0.0505 | 0.0451 |
| 64.75 | 0.0441 | 0.0487 | 0.0469 | 0.0455 | 0.0494 | 0.0463 |
| 65 | 0.0441 | 0.0494 | 0.0475 | 0.0457 | 0.0486 | 0.047 |
| 65.25 | 0.044 | 0.0502 | 0.0481 | 0.046 | 0.0477 | 0.0478 |
| 65.5 | 0.044 | 0.0503 | 0.0487 | 0.0463 | 0.0472 | 0.0487 |
| 65.75 | 0.0441 | 0.0495 | 0.048 | 0.0461 | 0.0464 | 0.0496 |
| 66 | 0.044 | 0.0485 | 0.0474 | 0.0459 | 0.0456 | 0.0504 |
| 66.25 | 0.044 | 0.0478 | 0.0469 | 0.0458 | 0.0449 | 0.0512 |
| 66.5 | 0.044 | 0.0471 | 0.0464 | 0.0456 | 0.0441 | 0.0518 |
| 66.75 | 0.044 | 0.0469 | 0.0457 | 0.0454 | 0.0435 | 0.0527 |
| 67 | 0.044 | 0.0477 | 0.0463 | 0.0456 | 0.0427 | 0.0532 |
| 67.25 | 0.044 | 0.0488 | 0.047 | 0.0457 | 0.0421 | 0.0539 |
| 67.5 | 0.044 | 0.0495 | 0.0476 | 0.0459 | 0.0428 | 0.0548 |
| 67.75 | 0.044 | 0.0505 | 0.0483 | 0.046 | 0.0439 | 0.0553 |
| 68 | 0.0439 | 0.0506 | 0.0489 | 0.0462 | 0.0448 | 0.0559 |
| 68.25 | 0.0439 | 0.0494 | 0.0482 | 0.0459 | 0.0459 | 0.0565 |
| 68.5 | 0.0439 | 0.0486 | 0.0474 | 0.0457 | 0.0467 | 0.057 |
| 68.75 | 0.0438 | 0.0476 | 0.0467 | 0.0454 | 0.0478 | 0.0579 |
| 69 | 0.0438 | 0.047 | 0.0458 | 0.0453 | 0.0484 | 0.0584 |
| 69.25 | 0.0438 | 0.0477 | 0.0464 | 0.0455 | 0.0492 | 0.059 |
| 69.5 | 0.0438 | 0.0489 | 0.047 | 0.0456 | 0.05 | 0.0593 |
| 69.75 | 0.0438 | 0.0496 | 0.0475 | 0.0458 | 0.0507 | 0.0586 |
| 70 | 0.0438 | 0.0503 | 0.0481 | 0.0459 | 0.0515 | 0.0575 |
| 70.25 | 0.0438 | 0.0504 | 0.0487 | 0.0461 | 0.0521 | 0.0563 |
| 70.5 | 0.0438 | 0.0497 | 0.0481 | 0.0459 | 0.0529 | 0.0555 |
| 70.75 | 0.0438 | 0.0486 | 0.0474 | 0.0457 | 0.0534 | 0.0544 |
| 71 | 0.0438 | 0.0479 | 0.0468 | 0.0455 | 0.054 | 0.0537 |
| 71.25 | 0.0438 | 0.0472 | 0.0461 | 0.0453 | 0.0533 | 0.0528 |
| 71.5 | 0.0438 | 0.0467 | 0.0455 | 0.0451 | 0.0525 | 0.0519 |
| 71.75 | 0.0438 | 0.0478 | 0.0462 | 0.0453 | 0.0514 | 0.051 |
| 72 | 0.0438 | 0.0486 | 0.0469 | 0.0456 | 0.0506 | 0.0502 |
| 72.25 | 0.0438 | 0.0496 | 0.0475 | 0.0458 | 0.0496 | 0.0496 |
| 72.5 | 0.0438 | 0.0503 | 0.0482 | 0.0461 | 0.0487 | 0.0488 |
| 72.75 | 0.0438 | 0.0505 | 0.0489 | 0.0463 | 0.0479 | 0.048 |
| 73 | 0.0438 | 0.0497 | 0.0482 | 0.0461 | 0.0473 | 0.0473 |
| 73.25 | 0.0438 | 0.0487 | 0.0475 | 0.0459 | 0.0465 | 0.0465 |
| 73.5 | 0.0438 | 0.048 | 0.0468 | 0.0456 | 0.0457 | 0.0459 |
| 73.75 | 0.0438 | 0.047 | 0.0461 | 0.0454 | 0.045 | 0.045 |
| 74 | 0.0438 | 0.0466 | 0.0454 | 0.0452 | 0.0442 | 0.0445 |
| 74.25 | 0.0438 | 0.0478 | 0.0461 | 0.0454 | 0.0436 | 0.0439 |
| 74.5 | 0.0438 | 0.0486 | 0.0468 | 0.0456 | 0.0428 | 0.0431 |
| 74.75 | 0.0438 | 0.0496 | 0.0474 | 0.0457 | 0.0422 | 0.0425 |
| 75 | 0.0438 | 0.0504 | 0.0481 | 0.0459 | 0.0429 | 0.0419 |
| 75.25 | 0.0438 | 0.0505 | 0.0488 | 0.0461 | 0.044 | 0.0414 |
| 75.5 | 0.0438 | 0.0497 | 0.0481 | 0.0459 | 0.0448 | 0.0405 |
| 75.75 | 0.0438 | 0.0486 | 0.0475 | 0.0457 | 0.046 | 0.0403 |
| 76 | 0.0438 | 0.048 | 0.0468 | 0.0455 | 0.0467 | 0.0419 |
| 76.25 | 0.0438 | 0.0472 | 0.0462 | 0.0453 | 0.0478 | 0.0429 |
| 76.5 | 0.0438 | 0.0467 | 0.0455 | 0.0451 | 0.0486 | 0.044 |
| 76.75 | 0.0438 | 0.0478 | 0.0462 | 0.0454 | 0.0495 | 0.0452 |
| 77 | 0.0438 | 0.0486 | 0.0469 | 0.0456 | 0.05 | 0.0461 |
| 77.25 | 0.0438 | 0.0496 | 0.0477 | 0.0459 | 0.0509 | 0.0472 |
| 77.5 | 0.0438 | 0.0503 | 0.0484 | 0.0461 | 0.0514 | 0.0479 |
| 77.75 | 0.0438 | 0.0508 | 0.0491 | 0.0464 | 0.0523 | 0.0488 |
| 78 | 0.0438 | 0.0499 | 0.0484 | 0.0462 | 0.0528 | 0.0497 |
| 78.25 | 0.0438 | 0.0488 | 0.0476 | 0.0461 | 0.0534 | 0.0503 |
| 78.5 | 0.0438 | 0.0481 | 0.0469 | 0.0459 | 0.054 | 0.0512 |
| 78.75 | 0.0438 | 0.0473 | 0.0461 | 0.0458 | 0.0533 | 0.0521 |
| 79 | 0.0438 | 0.0465 | 0.0454 | 0.0452 | 0.0525 | 0.0526 |
| 79.25 | 0.0438 | 0.0478 | 0.0462 | 0.0455 | 0.0513 | 0.0535 |
| 79.5 | 0.0438 | 0.0486 | 0.0469 | 0.0457 | 0.0506 | 0.054 |
| 79.75 | 0.0438 | 0.0496 | 0.0477 | 0.046 | 0.0496 | 0.0546 |
| 80 | 0.0438 | 0.0503 | 0.0484 | 0.0462 | 0.0488 | 0.0554 |
| 80.25 | 0.0438 | 0.0508 | 0.0492 | 0.0465 | 0.0479 | 0.056 |
| 80.5 | 0.0438 | 0.0499 | 0.0484 | 0.0463 | 0.0474 | 0.0566 |
| 80.75 | 0.0438 | 0.0488 | 0.0477 | 0.0461 | 0.0465 | 0.0571 |
| 81 | 0.0438 | 0.0481 | 0.0469 | 0.0458 | 0.0457 | 0.0577 |
| 81.25 | 0.0438 | 0.0471 | 0.0462 | 0.0456 | 0.0451 | 0.0583 |
| 81.5 | 0.0438 | 0.0466 | 0.0454 | 0.0454 | 0.0442 | 0.0591 |
| 81.75 | 0.0438 | 0.0478 | 0.0462 | 0.0457 | 0.0436 | 0.0594 |
| 82 | 0.0438 | 0.0486 | 0.047 | 0.0459 | 0.0423 | 0.0595 |
| 82.25 | 0.0438 | 0.0496 | 0.0478 | 0.0462 | 0.0427 | 0.0584 |
| 82.5 | 0.0438 | 0.0503 | 0.0486 | 0.0464 | 0.0441 | 0.0576 |
| 82.75 | 0.0438 | 0.051 | 0.0494 | 0.0467 | 0.0449 | 0.0564 |
| 83 | 0.0438 | 0.0497 | 0.0486 | 0.0465 | 0.0461 | 0.0556 |
| 83.25 | 0.0438 | 0.049 | 0.0478 | 0.0463 | 0.0468 | 0.0545 |
| 83.5 | 0.0438 | 0.048 | 0.047 | 0.046 | 0.0476 | 0.0538 |
| 83.75 | 0.0438 | 0.0473 | 0.0463 | 0.0458 | 0.0484 | 0.0529 |
| 84 | 0.0438 | 0.0467 | 0.0455 | 0.0456 | 0.0493 | 0.052 |
| 84.25 | 0.0438 | 0.0476 | 0.0463 | 0.0458 | 0.0499 | 0.0512 |
| 84.5 | 0.0438 | 0.0488 | 0.047 | 0.046 | 0.0508 | 0.0503 |
| 84.75 | 0.0438 | 0.0495 | 0.0478 | 0.0462 | 0.0513 | 0.0495 |
| 85 | 0.0438 | 0.0505 | 0.0485 | 0.0464 | 0.0522 | 0.0489 |
| 85.25 | 0.0438 | 0.0509 | 0.0493 | 0.0466 | 0.0527 | 0.0481 |
| 85.5 | 0.0438 | 0.0498 | 0.0485 | 0.0464 | 0.0536 | 0.0471 |
| 85.75 | 0.0438 | 0.049 | 0.0477 | 0.0462 | 0.0541 | 0.0466 |
| 86 | 0.0438 | 0.048 | 0.0469 | 0.046 | 0.0535 | 0.0457 |
| 86.25 | 0.0438 | 0.0473 | 0.0461 | 0.0458 | 0.0523 | 0.0452 |
| 86.5 | 0.0438 | 0.0465 | 0.0453 | 0.0456 | 0.0515 | 0.0443 |
| 86.75 | 0.0438 | 0.0478 | 0.0461 | 0.0458 | 0.0505 | 0.0438 |
| 87 | 0.0438 | 0.0485 | 0.0469 | 0.046 | 0.0497 | 0.0432 |
| 87.25 | 0.0438 | 0.0496 | 0.0476 | 0.0461 | 0.049 | 0.0426 |
| 87.5 | 0.0438 | 0.0503 | 0.0484 | 0.0463 | 0.0481 | 0.0418 |
| 87.75 | 0.0438 | 0.0508 | 0.0492 | 0.0465 | 0.0473 | 0.0413 |
| 88 | 0.0438 | 0.0499 | 0.0484 | 0.0463 | 0.0464 | 0.0407 |
| 88.25 | 0.0438 | 0.0488 | 0.0476 | 0.0461 | 0.0456 | 0.0402 |
| 88.5 | 0.0438 | 0.0481 | 0.0468 | 0.046 | 0.045 | 0.0418 |
| 88.75 | 0.0438 | 0.0471 | 0.0461 | 0.0458 | 0.0441 | 0.0427 |
| 89 | 0.0438 | 0.0466 | 0.0452 | 0.0456 | 0.0436 | 0.0441 |
| 89.25 | 0.0438 | 0.0476 | 0.0461 | 0.0459 | 0.043 | 0.0449 |
| 89.5 | 0.0438 | 0.0484 | 0.0469 | 0.0461 | 0.0421 | 0.0461 |
| 89.75 | 0.0438 | 0.0495 | 0.0478 | 0.0464 | 0.0428 | 0.0469 |
| 90 | 0.0438 | 0.0502 | 0.0486 | 0.0466 | 0.0439 | 0.0479 |
| 90.25 | 0.0438 | 0.051 | 0.0495 | 0.0469 | 0.0447 | 0.0485 |
| 90.5 | 0.0438 | 0.0499 | 0.0487 | 0.0467 | 0.0459 | 0.0494 |
| 90.75 | 0.0438 | 0.0488 | 0.0479 | 0.0465 | 0.0466 | 0.0502 |
| 91 | 0.0438 | 0.0481 | 0.047 | 0.0463 | 0.0477 | 0.051 |
| 91.25 | 0.0438 | 0.0471 | 0.0462 | 0.0461 | 0.0485 | 0.0516 |
| 91.5 | 0.0438 | 0.0466 | 0.0454 | 0.0459 | 0.0494 | 0.0525 |
| 91.75 | 0.0438 | 0.0476 | 0.0462 | 0.046 | 0.0499 | 0.053 |
| 92 | 0.0438 | 0.0484 | 0.0469 | 0.0461 | 0.0508 | 0.0539 |
| 92.25 | 0.0438 | 0.0494 | 0.0477 | 0.0463 | 0.0514 | 0.0545 |
| 92.5 | 0.0438 | 0.0501 | 0.0484 | 0.0464 | 0.0522 | 0.0551 |
| 92.75 | 0.0438 | 0.0508 | 0.0492 | 0.0465 | 0.0527 | 0.0556 |
| 93 | 0.0438 | 0.0498 | 0.0484 | 0.0463 | 0.0533 | 0.0562 |
| 93.25 | 0.0438 | 0.0487 | 0.0477 | 0.0461 | 0.0539 | 0.0568 |
| 93.5 | 0.0438 | 0.048 | 0.0469 | 0.0459 | 0.0532 | 0.0576 |
| 93.75 | 0.0438 | 0.047 | 0.0462 | 0.0457 | 0.0524 | 0.0582 |
| 94 | 0.0438 | 0.0466 | 0.0454 | 0.0455 | 0.0512 | 0.0587 |
| 94.25 | 0.0438 | 0.0475 | 0.0462 | 0.0457 | 0.0505 | 0.059 |
| 94.5 | 0.0438 | 0.0483 | 0.047 | 0.046 | 0.0495 | 0.0591 |
| 94.75 | 0.0438 | 0.0494 | 0.0477 | 0.0462 | 0.0487 | 0.0584 |
| 95 | 0.0438 | 0.0501 | 0.0485 | 0.0465 | 0.0478 | 0.0572 |
| 95.25 | 0.0438 | 0.0509 | 0.0493 | 0.0467 | 0.0473 | 0.056 |
| 95.5 | 0.0438 | 0.0498 | 0.0485 | 0.0465 | 0.0464 | 0.0552 |
| 95.75 | 0.0438 | 0.0487 | 0.0477 | 0.0463 | 0.0457 | 0.0542 |
| 96 | 0.0438 | 0.048 | 0.0469 | 0.046 | 0.0447 | 0.0534 |
| 96.25 | 0.0438 | 0.0473 | 0.0461 | 0.0458 | 0.0442 | 0.0525 |
| 96.5 | 0.0438 | 0.0465 | 0.0453 | 0.0456 | 0.0433 | 0.0516 |
| 96.75 | 0.0438 | 0.0472 | 0.0461 | 0.0458 | 0.0428 | 0.0507 |
| 97 | 0.0438 | 0.0484 | 0.0468 | 0.0459 | 0.0419 | 0.0499 |
| 97.25 | 0.0438 | 0.0492 | 0.0476 | 0.0461 | 0.0425 | 0.0493 |
| 97.5 | 0.0438 | 0.0502 | 0.0483 | 0.0462 | 0.0439 | 0.0485 |
| 97.75 | 0.0438 | 0.0507 | 0.0491 | 0.0464 | 0.0447 | 0.0476 |
| 98 | 0.0438 | 0.0498 | 0.0483 | 0.0462 | 0.0459 | 0.047 |
| 98.25 | 0.0438 | 0.0491 | 0.0476 | 0.046 | 0.0466 | 0.0461 |
| 98.5 | 0.0438 | 0.048 | 0.0468 | 0.0458 | 0.0474 | 0.0456 |
| 98.75 | 0.0438 | 0.0473 | 0.0461 | 0.0456 | 0.0482 | 0.0447 |
| 99 | 0.0438 | 0.0465 | 0.0453 | 0.0454 | 0.0491 | 0.0442 |
| 99.25 | 0.0438 | 0.0475 | 0.0461 | 0.0456 | 0.0499 | 0.0434 |
| 99.5 | 0.0438 | 0.0483 | 0.0469 | 0.0459 | 0.0505 | 0.0428 |
| 99.75 | 0.0438 | 0.0493 | 0.0476 | 0.0461 | 0.0514 | 0.0423 |
| 100 | 0.0438 | 0.05 | 0.0484 | 0.0464 | 0.0519 | 0.0416 |
| 100.25 | 0.0438 | 0.0508 | 0.0492 | 0.0466 | 0.0525 | 0.0408 |
| 100.5 | 0.0438 | 0.0498 | 0.0486 | 0.0464 | 0.0533 | 0.0403 |
| 100.75 | 0.0438 | 0.049 | 0.0479 | 0.0462 | 0.0539 | 0.04 |
| 101 | 0.0438 | 0.048 | 0.0473 | 0.0461 | 0.0531 | 0.0413 |
| 101.25 | 0.0438 | 0.047 | 0.0466 | 0.0458 | 0.0522 | 0.0427 |
| 101.5 | 0.0438 | 0.0473 | 0.0459 | 0.0457 | 0.0511 | 0.0439 |
| 101.75 | 0.0438 | 0.0465 | 0.0453 | 0.0455 | 0.0504 | 0.0447 |
| 102 | 0.0438 | 0.0474 | 0.0461 | 0.0457 | 0.0493 | 0.0459 |
| 102.25 | 0.0438 | 0.0482 | 0.0469 | 0.0459 | 0.0485 | 0.0467 |
| 102.5 | 0.0438 | 0.0493 | 0.0476 | 0.0461 | 0.0477 | 0.0478 |
| 102.75 | 0.0438 | 0.05 | 0.0484 | 0.0463 | 0.0471 | 0.0484 |
| 103 | 0.0438 | 0.0508 | 0.0492 | 0.0465 | 0.0463 | 0.0483 |
| 103.25 | 0.0438 | 0.0497 | 0.0484 | 0.0463 | 0.0455 | 0.0493 |
| 103.5 | 0.0438 | 0.049 | 0.0476 | 0.046 | 0.0446 | 0.0501 |
| 103.75 | 0.0438 | 0.0479 | 0.0468 | 0.0458 | 0.0441 | 0.0509 |
| 104 | 0.0438 | 0.0472 | 0.046 | 0.0456 | 0.0432 | 0.0515 |
| 104.25 | 0.0438 | 0.0464 | 0.0452 | 0.0457 | 0.0427 | 0.0523 |
| 104.5 | 0.0438 | 0.0471 | 0.046 | 0.0459 | 0.0418 | 0.0529 |
| 104.75 | 0.0438 | 0.0483 | 0.0467 | 0.046 | 0.0426 | 0.0538 |
| 105 | 0.0438 | 0.049 | 0.0475 | 0.0461 | 0.0437 | 0.0544 |
| 105.25 | 0.0438 | 0.05 | 0.0482 | 0.0463 | 0.0445 | 0.055 |
| 105.5 | 0.0438 | 0.0506 | 0.049 | 0.0464 | 0.0457 | 0.0555 |
| 105.75 | 0.0438 | 0.0499 | 0.0483 | 0.0462 | 0.0464 | 0.0564 |
| 106 | 0.0438 | 0.0487 | 0.0475 | 0.046 | 0.0475 | 0.0569 |
| 106.25 | 0.0438 | 0.048 | 0.0468 | 0.0458 | 0.0483 | 0.0575 |
| 106.5 | 0.0437 | 0.0473 | 0.046 | 0.0456 | 0.0491 | 0.058 |
| 106.75 | 0.0437 | 0.0465 | 0.0453 | 0.0454 | 0.0497 | 0.0586 |
| 107 | 0.0437 | 0.0474 | 0.0461 | 0.0456 | 0.0506 | 0.0592 |
| 107.25 | 0.0437 | 0.0482 | 0.0469 | 0.0458 | 0.0511 | 0.0595 |
| 107.5 | 0.0437 | 0.0492 | 0.0476 | 0.0461 | 0.0518 | 0.0582 |
| 107.75 | 0.0437 | 0.0499 | 0.0484 | 0.0463 | 0.0526 | 0.0574 |
| 108 | 0.0437 | 0.0508 | 0.0492 | 0.0465 | 0.0532 | 0.0562 |
| 108.25 | 0.0437 | 0.0497 | 0.0484 | 0.0463 | 0.0537 | 0.0554 |
| 108.5 | 0.0437 | 0.049 | 0.0477 | 0.0461 | 0.053 | 0.0543 |
| 108.75 | 0.0437 | 0.0479 | 0.0469 | 0.0459 | 0.0522 | 0.0535 |
| 109 | 0.0437 | 0.0472 | 0.0462 | 0.0457 | 0.051 | 0.0527 |
| 109.25 | 0.0437 | 0.0465 | 0.0454 | 0.0455 | 0.0503 | 0.0518 |
| 109.5 | 0.0437 | 0.0473 | 0.0461 | 0.0457 | 0.0492 | 0.0509 |
| 109.75 | 0.0437 | 0.048 | 0.0468 | 0.0458 | 0.0487 | 0.05 |
| 110 | 0.0437 | 0.0491 | 0.0476 | 0.046 | 0.0478 | 0.0492 |
| 110.25 | 0.0437 | 0.0498 | 0.0483 | 0.0461 | 0.047 | 0.0484 |
| 110.5 | 0.0437 | 0.0506 | 0.049 | 0.0463 | 0.0462 | 0.0479 |
| 110.75 | 0.0437 | 0.0498 | 0.0483 | 0.0461 | 0.0453 | 0.047 |
| 111 | 0.0437 | 0.0487 | 0.0475 | 0.0459 | 0.0447 | 0.0464 |
| 111.25 | 0.0437 | 0.048 | 0.0468 | 0.0457 | 0.0439 | 0.0455 |
| 111.5 | 0.0437 | 0.047 | 0.046 | 0.0455 | 0.0433 | 0.045 |
| 111.75 | 0.0437 | 0.0465 | 0.0453 | 0.0453 | 0.0425 | 0.0441 |
| 112 | 0.0437 | 0.0473 | 0.046 | 0.0455 | 0.0419 | 0.0436 |
| 112.25 | 0.0437 | 0.0481 | 0.0467 | 0.0457 | 0.0423 | 0.0427 |
| 112.5 | 0.0437 | 0.0492 | 0.0475 | 0.0459 | 0.0437 | 0.0422 |
| 112.75 | 0.0437 | 0.0499 | 0.0482 | 0.0461 | 0.0445 | 0.0416 |
| 113 | 0.0437 | 0.0506 | 0.0489 | 0.0463 | 0.0457 | 0.0411 |
| 113.25 | 0.0437 | 0.0498 | 0.0482 | 0.0461 | 0.0464 | 0.0404 |
| 113.5 | 0.0437 | 0.0487 | 0.0475 | 0.0459 | 0.0474 | 0.0399 |
| 113.75 | 0.0437 | 0.048 | 0.0468 | 0.0458 | 0.048 | 0.0412 |
| 114 | 0.0437 | 0.047 | 0.0461 | 0.0456 | 0.0488 | 0.0425 |
| 114.25 | 0.0437 | 0.0465 | 0.0454 | 0.0454 | 0.0496 | 0.0434 |
| 114.5 | 0.0437 | 0.0473 | 0.0461 | 0.0456 | 0.0505 | 0.0447 |
| 114.75 | 0.0437 | 0.048 | 0.0468 | 0.0458 | 0.051 | 0.0455 |
| 115 | 0.0437 | 0.049 | 0.0476 | 0.046 | 0.0519 | 0.0466 |
| 115.25 | 0.0437 | 0.0497 | 0.0483 | 0.0462 | 0.0524 | 0.0474 |
| 115.5 | 0.0437 | 0.0505 | 0.049 | 0.0464 | 0.053 | 0.0483 |
| 115.75 | 0.0437 | 0.0498 | 0.0481 | 0.0462 | 0.0536 | 0.0492 |
| 116 | 0.0437 | 0.049 | 0.0473 | 0.046 | 0.0532 | 0.05 |
| 116.25 | 0.0437 | 0.048 | 0.0466 | 0.0458 | 0.0521 | 0.0506 |
| 116.5 | 0.0437 | 0.0473 | 0.0458 | 0.0456 | 0.0513 | 0.0515 |
| 116.75 | 0.0437 | 0.0462 | 0.045 | 0.0454 | 0.0502 | 0.052 |
| 117 | 0.0437 | 0.047 | 0.0458 | 0.0456 | 0.0495 | 0.0526 |
| 117.25 | 0.0437 | 0.0482 | 0.0465 | 0.0457 | 0.0487 | 0.0535 |
| 117.5 | 0.0437 | 0.0489 | 0.0473 | 0.0458 | 0.0478 | 0.054 |
| 117.75 | 0.0437 | 0.0499 | 0.048 | 0.046 | 0.047 | 0.0547 |
| 118 | 0.0437 | 0.0504 | 0.0488 | 0.0462 | 0.0462 | 0.0555 |
| 118.25 | 0.0437 | 0.0498 | 0.0481 | 0.046 | 0.0454 | 0.056 |
| 118.5 | 0.0437 | 0.049 | 0.0474 | 0.0458 | 0.0448 | 0.0566 |
| 118.75 | 0.0437 | 0.048 | 0.0467 | 0.0456 | 0.0439 | 0.0571 |
| 119 | 0.0437 | 0.0473 | 0.046 | 0.0454 | 0.0433 | 0.0577 |
| 119.25 | 0.0437 | 0.0465 | 0.0453 | 0.0452 | 0.0425 | 0.0583 |
| 119.5 | 0.0437 | 0.0471 | 0.046 | 0.0454 | 0.0419 | 0.0588 |
| 119.75 | 0.0437 | 0.0479 | 0.0468 | 0.0457 | 0.0424 | 0.0592 |
| 120 | 0.0437 | 0.049 | 0.0475 | 0.0459 | 0.0435 | 0.0579 |
